# Discordant Gene Expression Signatures and Related Phenotypic Differences in Lamin A- and A/C-Related Hutchinson-Gilford Progeria Syndrome (HGPS)

**DOI:** 10.1371/journal.pone.0021433

**Published:** 2011-06-27

**Authors:** Martina Plasilova, Chandon Chattopadhyay, Apurba Ghosh, Friedel Wenzel, Philippe Demougin, Christoph Noppen, Nathalie Schaub, Gabor Szinnai, Luigi Terracciano, Karl Heinimann

**Affiliations:** 1 Research Group Human Genetics, Department of Biomedicine, University of Basel, and Division of Medical Genetics, University Children's Hospital, Basel, Switzerland; 2 Institute of Child Health, Kolkata, India; 3 S.B. Devi Charity Home, Kolkata, India; 4 Life Sciences Training Facility and Division of Molecular Psychology, Biozentrum and Pharmazentrum, University of Basel, Basel, Switzerland; 5 Viollier AG, Basel, Switzerland; 6 Division of Pediatric Endocrinology/Diabetology, University Children's Hospital Basel, and Department of Biomedicine, University of Basel, Basel, Switzerland; 7 Institute for Pathology, University of Basel, Basel, Switzerland; National Institute of Allergy and Infectious Diseases, United States of America

## Abstract

Hutchinson-Gilford progeria syndrome (HGPS) is a genetic disorder displaying features reminiscent of premature senescence caused by germline mutations in the *LMNA* gene encoding lamin A and C, essential components of the nuclear lamina. By studying a family with homozygous *LMNA* mutation (K542N), we showed that HGPS can also be caused by mutations affecting both isoforms, lamin A and C. Here, we aimed to elucidate the molecular mechanisms underlying the pathogenesis in both, lamin A- (sporadic) and lamin A and C-related (hereditary) HGPS. For this, we performed detailed molecular studies on primary fibroblasts of hetero- and homozygous *LMNA* K542N mutation carriers, accompanied with clinical examinations related to the molecular findings. By assessing global gene expression we found substantial overlap in altered transcription profiles (13.7%; 90/657) in sporadic and hereditary HGPS, with 83.3% (75/90) concordant and 16.7% (15/90) discordant transcriptional changes. Among the concordant ones we observed down-regulation of *TWIST2*, whose inactivation in mice and humans leads to loss of subcutaneous fat and dermal appendages, and loss of expression in dermal fibroblasts and periadnexial cells from a *LMNA* ^K542N/K542N^ patient further confirming its pivotal role in skin development. Among the discordant transcriptional profiles we identified two key mediators of vascular calcification and bone metabolism, *ENPP1* and *OPG*, which offer a molecular explanation for the major phenotypic differences in vascular and bone disease in sporadic and hereditary HGPS. Finally, this study correlates reduced *TWIST2* and *OPG* expression with increased osteocalcin levels, thereby linking altered bone remodeling to energy homeostasis in hereditary HGPS.

## Introduction

Hutchinson-Gilford progeria syndrome (HGPS) is a genetic disorder caused by mutations in the lamin A/C gene (*LMNA*), a component of the nuclear lamina [1]. Within the broad spectrum of phenotypes caused by *LMNA* germline mutations (known as laminopathies), HGPS belongs to the distinct group of segmental progeroid syndromes, displaying features reminiscent of premature senescence [2], [3]. The main tissues affected in HGPS are of mesenchymal origin, and include adipose tissue, bone, cartilage and the cardiovascular system. Progeria is a progressive disease: Affected children appear normal at birth, but begin to develop characteristic symptoms within the first years of life. The main symptoms of HGPS include growth retardation, generalized lipodystrophy (cachexia), osteoporosis and osteolysis, decreased joint mobility, joint stiffness, skin atrophy, hair loss and cardiovascular changes resulting in death on average at 12 to 13 years of age [4], [5].

The *LMNA* gene encodes two A-type lamins, lamin A and C, which are the result of alternative splicing. Generated lamin A and C share the first 566 amino acids and differ by the 98 and 6 amino acids at their C-terminal end, respectively. Pre-lamin A, but not lamin C, is subjected to several posttranslational modifications, during which its C terminus is modified by farnesylation, followed by endoproteolytic cleavage by the Zmpste24 protease [6]. The A-type lamins, together with B-type lamins, are type V intermediate filament proteins that form a filamentous meshwork underlying the inner membrane of the nuclear envelope, known as the nuclear lamina. Through their direct or indirect interaction with many known nuclear membrane and nucleoplasmic proteins lamins were shown to be involved in a number of essential nuclear functions, including maintenance of nuclear integrity, DNA replication, transcription organization, replication, and DNA repair [7], [8], [9]. In contrast to B-type lamins, which are ubiquitously expressed in all cell types at all developmental stages [7], [10], [11], A-type lamins are expressed in differentiated tissues, mesenchymal and hair stem cells, but are absent in other types of stem cells, including embryonic stem cells, and exist at very low level or are absent in hematopoietic cells [12], [13], [14], [15].

The vast majority of HGPS patients are sporadic cases caused by a *de novo* heterozygous germline mutation c.1824C > T (p.G608G) which generates a cryptic splice site in exon 11 of *LMNA,* and leads to an in-frame deletion of 50 amino acids in pre-lamin A [16], [17]. The mutant protein, so called “progerin“, lacks the cleavage site for the enzyme Zmpste24, thus preventing the final cleavage step in the pre-lamin A posttranslational processing. As a consequence, lamin A remains permanently carboxyfarnesylated and methylated, which leads to its abnormal incorporation into the nuclear lamina and thickening of the nuclear lamina and a large spectrum of nuclear abnormalities [18], [19], [20], [21].

Initially it was thought that HGPS is merely a lamin A-related laminopathy, caused by constitutive production of progerin. By studying a HGPS family with parental consanguinity, our research group was the first to provide evidence that HGPS can also be caused by homozygous mutations (c.1626G>C; p.K542N) affecting both, lamin A and C, thus challenging the prevailing hypothesis that HGPS merely represents a lamin A-related laminopathy [4]. This observation was further supported by the identification of other lamin A/C-related *LMNA* mutations in patients with progeroid disorders [22], [23], [24], showing that progerin or pre-lamin A accumulation is not the major determinant of the progeroid phenotype.

In order to elucidate the molecular mechanisms underlying the pathogenesis in both, lamin A- (sporadic) and lamin A/C-related (hereditary) HGPS, we performed detailed molecular studies on primary fibroblasts of hetero- and homozygous K542N mutation carriers, accompanied with clinical examinations related to the molecular findings. Here, we show that there is substantial overlap in altered gene expression profiles between G608G- (sporadic) and K542N-related (hereditary) HGPS. The concordant as well as the discordant transcriptional changes point to common pathogenic processes underlying both types of HGPS and offer molecular explanations for the major differences in disease expression, namely bone and cardiovascular disease as well as altered energy homeostasis.

## Results

### Molecular characterization of *LMNA* K542N

In contrast to the lamin A specific G608G mutation, the homozygous *LMNA* missense mutation (c.1626G>C, p.K542N) identified in the consanguineous HGPS family alters the coding sequence shared by both splice variants and thus affects both, lamin A and C. RT-PCR analysis of RNA extracted from cultured fibroblasts of two K542N homozygous patients, healthy heterozygous parents and sister confirmed, as predicted by *in silico* splice site analysis [4], that the c.1626G>C mutation neither affects lamin A/C mRNA splicing nor introduces a novel splice site (Figure S1). To assess whether K542N affects expression and processing of A-type lamins, we undertook Western blot analysis of total protein extracts from fibroblasts using lamin A specific (133A2), and lamin A and C specific antibodies (JOL5). Western blots neither revealed any difference in lamin A and C expression patterns nor gave evidence for prelamin A or aberrant sized lamin A/C accumulation in patients (n = 2) when compared with healthy family members (n = 3) and control (Figure S2). This observation is consistent with our previous assumption that K542N does not affect lamin A processing since the mutation is localized outside the region recognized by Rce1 and Zmpste24, enzymes involved in posttranslational modifications [4].

### Nuclear abnormalities in *LMNA* K542N fibroblasts

**Figure 1:**
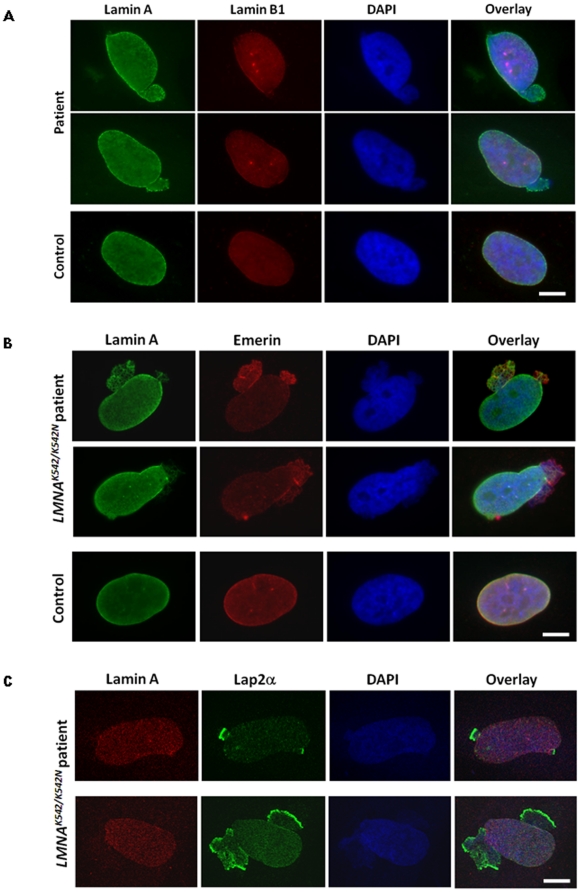
Fibroblast nuclei from the *LMNA^K542/K542N^* patient and healthy control. (A) Immunofluorescence staining for lamin A and lamin B1. (B) Immunofluorescence microscopy using lamin A and emerin antibodies. (C) Confocal microscopy using lamin A and LAP2 antibodies. Scale bar, 10 µM.

Cultured cells from sporadic HGPS patients carrying the progerin mutation were shown to exhibit a variety of alterations in nuclear morphology [18], [19], [20], [21]. To assess the nuclear phenotype in K542N mutation carriers we examined primary cultured fibroblasts from affected and healthy family members by immunofluorescence. The nuclei of K542N carriers showed one or two extensive protrusions frequently present at opposite poles, accompanied with honeycomb and/or tubule-like lamin structures. DAPI counterstaining of DNA revealed that some of the protrusions show envelope rupture accompanied by chromatin extrusion (Figure 1). These nuclear abnormalities were present in approximately 28% of nuclei from homozygous K542N mutation carriers (n = 3) and in 5% of nuclei from healthy heterozygous carriers (n = 3; 28% *vs*. 5%, p<0.0001). Control fibroblasts (n = 3) showed a similar percentage of nuclear envelope alterations (3%) as observed in the heterozygotes, but the morphological changes were different from those observed in K542N carriers and neither chromatin extrusion nor honeycomb structures were displayed. Lamin B1 stain was found to be reduced to absence in nearly all patient's nuclear protrusions (Figure 1A). Immunostaining for emerin and LAP2, whose interaction with A-type lamins is expected to be impaired by K542N, both showed abnormal localization within these protrusions. Increased emerin presence was detected in the majority of nuclear protrusions. In the large blebs, emerin showed the honeycomb pattern, which frequently overlapped with that of lamin A (Figure 1B). Interestingly, striking LAP2 accumulation was identified at the frontal edge of nearly all nuclear protrusions even though lamin A was frequently absent in these regions (Figure 1C). These observations support the assumption that the K542N mutation impairs the interaction of the A-type lamins with LAP2 [4]. In particular, failure of the LAP2 protein to colocalize with lamin A points to a derangement of lamin A-LAP2 complexes by K542N [25].

**Figure 2:**
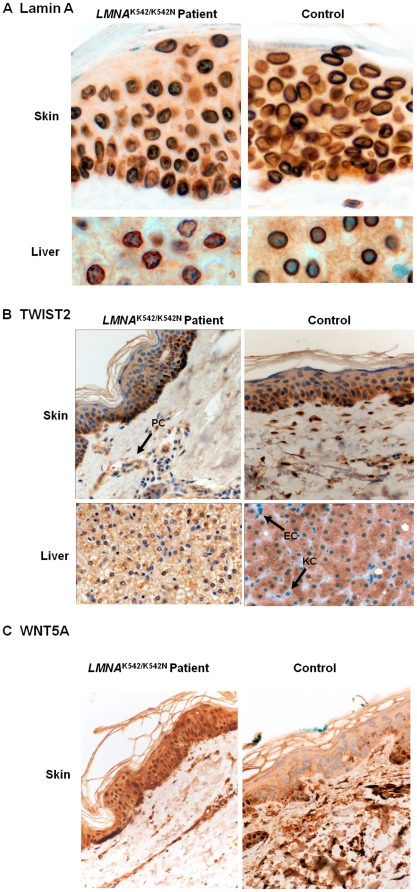
Immunohistochemical analysis in skin and liver autoptic specimens from a deceased *LMNA^K542/K542N^* patient. (A) Immunohistochemistry for lamin A. (B) Immunohistochemical staining for Twist2. Arrow indicates Twist2 loss in the periadenexial cells (PC) of the dermis in the skin and in the Kupffer (KC) and endothelial cells (EC) of the liver. (C) Immunohistochemistry for Wnt5a. Scale bar, 10 µM.

To differentiate whether the misshapen nuclei are restricted to fibroblasts (or culture artifacts), we performed immunohistochemical (IHC) staining of lamin A in skin and liver autopsy specimens from a deceased homozygous K542N carrier. In contrast to control samples, the majority of patient's hepatocyte nuclei showed an irregular nuclear contour (32% *vs*. 4.1%, p<0.0001), i.e. wrinkled nuclear shape with protrusions and occasional spikes. IHC analysis of the skin specimen revealed similar, but less striking changes (Figure 2A). We thus provide direct evidence that nuclear envelope alterations, which have been considered a pathological hallmark of cultured HGPS cells [18], [20], [26], are already present *in vivo* [18], [20], [26], [27].

### Comparison of global gene expression in lamin A- and lamin A/C-related HGPS

With the aim to elucidate the molecular mechanisms underlying the pathogenesis in both, lamin A- (sporadic) and lamin A/C-related (hereditary) HGPS, we investigated primary cultured skin fibroblasts from affected homozygous K542N carriers (*LMNA^K542/K542N^*; n = 3), healthy heterozygotes (*LMNA^K542/+^*; n = 3), and controls (n = 3) for differences in global gene expression using GeneChip Human Genome U133 Plus 2.0 arrays (Affymetrix UK Ltd.). Comparison of the *LMNA^K542/K542N^* transcriptional profile with the ones from healthy carriers and controls revealed at least 1.5 fold expression changes in 657 and more than 2 fold changes in 278 genes (Table S1), from which a selection of 19 altered transcripts could be confirmed by subsequent TaqMan gene expression assays (Table S2). Expression changes ranged from −7.06 to +10.7 fold. Analysis of differentially expressed genes revealed that the K542N mutation leads to deregulation of genes involved in cell proliferation and differentiation pathways as well as in tissue development.

Since sporadic and hereditary HGPS show considerable clinical overlap we wondered about the degree of concordance in transcriptional signatures between K542N and G608G fibroblasts. Comparison of our results with the two published gene expression microarray experiments on fibroblast cell lines from patients carrying the G608G mutation revealed substantial overlap in altered transcription patterns (13.7%; 90/657) between sporadic and hereditary HGPS (Table 1). Of these, 78 genes overlapped with the study by Csoka et al. (86.6%, 78/90; [28]), 23 with study of Wang et al. (25.5%, 23/90; [29]), and 11 with both (12.2%; 11/90). Ten of them were subsequently confirmed by TaqMan expression assays (Table S2). Recently, the third global expression profiling on fibroblasts from patients with sporadic HGPS was reported [30], however, since the authors did not publish the list of differentially expressed genes, we could not include them in our comparative study.

**Table 1:** Overlap between *LMNA* K542N and G608G expression signatures.

	LMNA^K542/K542N^	LMNA^G608G/+^ [28]	LMNA^G608G/+^ [29]
*LMNA^K542/K542N^*	1	11.9% (78/657)	3.5% (23/657)
*LMNA^G608G/+^* [28]	21.6% (78/361)	1	5.5% (20/361)
*LMNA^G608G/+^* [29]	41.1% (23/56)	35.7% (20/56)	1

Comparison between transcriptional signature in *LMNA^K542/K542N^* fibroblasts and the two published transcriptional signatures in *LMNA^G608G/+^* fibroblast cell lines [28], [29]. Total number of gene transcripts compared: this study, n = 657, Csoka et al. [28]: n = 361, Wang et al. [29]: n = 56. Overall overlap between K542N and G608G expression signatures: 90 genes (13.7%; 90/657). Overlap between the two G608G expression signatures: 20 genes.

With regard to the overlapping genes, the transcriptional changes observed in *LMNA^G608G/+^* cell lines were usually higher, ranging from −6.8 to 29.1-fold and from -29.56 to 51.85-fold, respectively [28], [29], compared to *LMNA^K542/K542N^* fibroblasts (−6.46 to 7-fold). Twenty-seven of the overlapping genes (30.0%; 27/90) showed only 1.5–2 fold expression changes in *LMNA^K542/K542N^* cells, and therefore would have been missed applying a 2-fold change cutoff. These differences may partly reflect true differences related to the particular *LMNA* mutation, and/or be due to differences in methodology and passage number, as the *LMNA^G608G/+^* cells obtained from the Corriell Cell Repository are usually of later passages (>10) and the authors did not specify the passage number of the fibroblasts used [28], [29].

Analysis of the common transcriptional signatures of sporadic and hereditary HGPS fibroblasts revealed that 83.3% (75/90) of the genes had concordant (matching) and 16.7% (15/90) discordant (opposite) transcription patterns (Table S3A and S3B, respectively). Interestingly, the overlap between the G608G and K542N fibroblasts' gene expression profiles was higher than between the two G608G studies alone. Importantly, the differentially expressed genes common to all three studies showed matching transcriptional changes (Table 1 and Table S4).

### Concordant (matching) transcriptional signatures

One of the genes that showed concordant transcriptional alteration in both *LMNA^K542/K542N^* and *LMNA^G608G/+^* cells was *TWIST2* (also known as *DERMO1*), a known transcription factor important for osteoblast differentiation [31], whose knock-out mouse models display a cachectic phenotype similar to HGPS, i.e. postnatal failure to thrive, growth retardation, adipose deficiency, and perinatal death [32]. Analogous to cachectic mice, *TWIST2* expression was found to be 3 fold decreased in the *LMNA^G608G/+^* cells [28], and 3.5 fold decreased (p = 0.00011) in *LMNA^K542/K542N^* fibroblasts which was confirmed by two TaqMan expression assays (fold change of −3.2, p = 0.016, and of −2.9, p = 0.016).

To assess TWIST2 protein expression *in vivo* we investigated skin and liver autopsy specimens from a deceased K542N homozygous patient. Immunohistochemical analysis confirmed decreased cytoplasmic TWIST2 expression levels in the patient's hepatocytes when compared to corresponding specimens from healthy controls, and revealed loss of nuclear TWIST2 in the majority of patient's hepatocytes (Figure 2B). The absence of nuclear TWIST2 in Kupffer and endothelial cells in both patient's and control's liver specimens excluded the possibility of a technical artifact. In skin epidermis, TWIST2 staining did not reveal any apparent expression differences between patient and control. In the patient's dermis, however, selective loss of TWIST2 was detected in most periadnexial and fibroblast cells, which is in accordance with the decreased *TWIST2* transcription levels we observed in cultured fibroblasts by microarrays and TaqMan assays.

Because *Twist2* mutant mice also manifest abnormalities in glucose metabolism and storage, evidenced by hypoglycemia and absence of glycogen in skeletal muscle and liver, as well as by elevated transcription of proinflammatory cytokines (TNFα and IL-1β) in skin, skeletal muscle, and cultured skin fibroblasts [32], we wondered if *TWIST2* down-regulation could result in similar molecular and biochemical changes in our patient(s). Periodic acid-Schiff (PAS) staining for glycogen performed on the liver autoptic specimen from the deceased patient did not reveal any obvious quantitative changes (data not shown). Similarly, serum TNFα assessment in two homozygous patients and their heterozygous parents, as well as the microarray results on *TNFα* did not show any differences between affected and healthy family members (data not shown). Microarray analysis, however, revealed almost 50% up-regulation of IL-1β in patients' cultured fibroblasts (fold change = 1.41, p<0.01), the finding subsequently confirmed by TaqMan analysis (fold change of 4.38, p = 0.044, and of 4.50, p = 0.046). Glucose assessment, performed within oral glucose tolerance testing (oGTT; see below) in two patients and their parents, revealed a fasting glucose at the lower normal level in both patients (Table S5).

### Discordant (opposite) transcriptional signatures

Given that 16.7% (15/90) of the overlapping genes in sporadic and hereditary HGPS showed opposite transcriptional changes, we wondered whether some of these findings might help to explain differences in disease expression. Interestingly, two genes, *TNFRSF11B* and *ENPP1*, whose mutations are associated with distinct disease phenotypes present in HGPS were found to have opposite transcriptional changes in *LMNA^K542/K542N^* and *LMNA^G608G/+^* fibroblasts (Table S3B; [28]).

*TNFRSF11B*, also known as osteoprotegerin (*OPG*), is a member of the *OPG*/*RANKL*/*RANK* cytokine triad controlling osteoclastogenesis and bone remodeling, which was recently identified as a mediator of vascular calcification. Its increased serum levels were shown to be associated with vascular calcification, coronary artery disease, stroke and cardiovascular events [33], [34]. In microarray experiments *OPG* expression was increased 3 fold in *LMNA^G608G/+^* cell lines [28] and 2.3 fold decreased (p = 0.023) in the *LMNA^K542/K542N^* fibroblasts, which was also confirmed by two TaqMan assays (fold change of −2.27, p = 0.052, and of −2.41, p = 0.037). These findings thus demonstrate an inverse correlation between *OPG* expression and atherosclerotic heart disease in sporadic and hereditary HGPS.

In addition to *TNFRSF11B*, the cardiovascular phenotype in sporadic and hereditary HGPS also correlated with *ENPP1* expression. ENPP1, an ectonucleotide pyrophosphatase phosphodiesterase 1 (known also as plasma cell antigen 1, PC-1), is a widely expressed cell surface enzyme which generates inorganic pyrophosphate (PPi), a solute that serves as an essential physiologic inhibitor of calcification [35]. In addition, ENPP1 was shown to be an inhibitor of the insulin signaling pathway, through its direct interaction with the insulin receptor [36]. Consistently with its dual function, *ENPP1* inactivating mutations caused generalized arterial calcification of infancy (GACI; [35]), and over-expression of *ENPP1* has been found to be associated with human insulin resistance in non-insulin-dependent diabetes mellitus [36], [37]. Microarray experiments showed that *ENPP1* expression was 2.4 fold (p = 0.024) increased in *LMNA^K542/K542N^* fibroblasts and 6.8 fold decreased in *LMNA^G608G/+^* fibroblast cell lines [28]. TaqMan expression analysis in *LMNA^K542/K542N^* fibroblasts confirmed the *ENPP1* expression changes, but did not reach statistical significance (fold change of 3.00, p = 0.064, and of 3.32, p = 0.055).

Cardiological examination in three patients (aged 4–17 years at time of referral) was unremarkable. Systolic and diastolic blood pressures were within the lower normal age- and sex-related ranges (patient R: 95/65 mmHg; patient E: 100/70 mmHg; patient A: 95/65 mmHg). Pulse rates were at the upper normal levels (116–120/min). Electrocardiogram and echocardiographic investigations showed no pathological findings. Imaging studies (CT) of the heart and the large vessels gave no evidence for atherosclerotic alterations in any of the patients. These findings match with the cause of death observed in the hereditary HGPS patients. Unlike patients with sporadic HGPS in whom the cause of death is usually of vascular origin [2], [38], all deceased K542N homozygous patients, two previously reported (at age 10 and 19 years [4]) and two recently deceased (at age 21 and 21 years), died of respiratory failure due to severe pneumonia. The pulmonary infections resulted from hypoventilation due to progressive rib resorption and subsequent rib cage instability. Taken together, the normal cardiovascular findings in the three patients investigated are in accordance with increased *ENPP1* expression levels observed in the homozygous K542N fibroblasts. This observation is further supported by up-regulation of osteopontin (*OPN*, also known as secreted phosphoprotein 1, *SPP1*), whose expression in mice was shown to be positively regulated by *ENPP1*, through the inorganic pyrophosphate level [39]. Up-regulation of *SPP1* in *LMNA^K542/K542N^* fibroblasts was detected by both microarrays and TaqMan expression assay (fold change of 2.7, p = 0.0026, and of 14.2, p = 0061, respectively), thus further confirming the correlation of increased *ENPP1* expression levels with an unremarkable cardiovascular status in hereditary HGPS.

In an effort to clarify whether increased expression of *ENPP1* is associated with insulin resistance and diabetes in *LMNA* K542N homozygotes, oral glucose tolerance testing (oGTT) was performed on both healthy parents and two affected children (at age 10 and 20 years; Table S5B). In both homozygous patients glucose concentration at fasting (0 minutes of oGTT) and after glucose load (120 minutes of oGTT) was found to be at low normal level and did not increase postprandially, thus excluding diabetes mellitus type 2, impaired fasting glucose or impaired glucose tolerance. Fasting insulin levels were within the lower normal range and showed nearly no increase of insulin at 120 minutes after glucose load. Intriguingly, C-peptide measurements in the affected sibs were repeatedly found to be very low, with apparent age-dependent pattern (ranging from 0.24 to 0.08 nmol/L; Table S5), correlating with decreased insulin levels and suggesting increased insulin sensitivity. Nonlinear homeostatic model assessment (HOMA2; [40]), using two independent, one year consecutive measurements of fasting insulin, C-peptide, and fasting glucose, revealed decreased insulin resistance (HOMA2-IR<0.5), strongly increased insulin sensitivity (>240%), and decreased β–cell function (<77%) in *LMNA* K542N homozygotes (Table S5A). High insulin sensitivity was further confirmed by the insulin sensitivity indices derived from oGTT (Matsuda and Cederholm index, Table S5B; [41], [42], [43]). In contrast to the K542N homozygous children, the heterozygous parents met the criteria for impaired glucose tolerance (mother) and diabetes (father). They showed no insulin resistance (HOMA-IR = 1), normal to moderately increased insulin sensitivity (Matsuda and Cederholm index), but decreased β–cell response to glucose uptake (Table S5). To further investigate possible reasons for the observed increase in insulin sensitivity, we measured osteocalcin, an osteoblast-specific protein which acts as a hormone regulating insulin production and sensitivity [44]. Total serum osteocalcin levels were found to be elevated in both patients (106 ug/L [normal range at age 10y: 24.0–70.0] and 50.5 ug/L [normal range at age 20y: 14.0–42.0], respectively), but not in their parents. Taken together, *ENPP1* up-regulation observed in the homozygous patients is not associated with insulin resistance. Increased insulin sensitivity, however, observed in two consecutive laboratory investigations is in accordance with increased levels of total osteocalcin.

### Differentially expressed genes unique to *LMNA^K542/K542N^* fibroblasts

We realized that more than 200 of the differentially expressed genes encode proteins that are either secreted into extracellular environment or are known membrane anchored proteins affecting signaling of adjacent cells, indicating that deranged gene expression in fibroblasts might indirectly influence differentiation and activity of neighboring cells. Among these genes we identified the Wnt5a ligand, a known wnt secreted signaling protein involved in the cell-lineage decision of mesenchymal stem cells when they differentiate into adipocytes or osteoblasts [45]. *WNT5A* deregulation was exclusively observed in *LMNA^K542/K542N^* fibroblasts. Both, microarray and TaqMan gene expression analysis, showed up-regulation in K542N homozygous fibroblasts compared to heterozygous and control fibroblasts (fold change of 2.82, p = 0.00084; and of 3.98, p = 0.026, respectively). Immunohistochemical assessment of WNT5A expression on autopsy skin specimens from a homozygous K542N patient showed diffuse WNT5A expression in all four layers of the epidermis, in contrast to control skin where it was restricted to keratinocytes of the basal epidermal layer (Figure 2C).

## Discussion

This study provides the first detailed molecular biological analysis of lamin A and C-related, hereditary HGPS relating the transcriptional signature to the respective biochemical and phenotypic features and comparing it with lamin A-related, sporadic progeria. By investigating a consanguineous family with 4 affected children carrying a homozygous *LMNA* mutation (c.1626G>C; p.K542N) we previously provided evidence for an autosomal recessive form of HGPS, thus challenging the prevailing view that HGPS merely represents a sporadic autosomal dominant, lamin A-related laminopathy. Here we confirm our previous assumption [4], that K542N does neither affect *LMNA* mRNA splicing nor lamin A processing. Meanwhile, similar observations have been reported in a 2-year-old HGPS patient with age-pronounced acro-osteolysis who carried compound heterozygous mutations, p.T528M and p.M540T, localized in the C-terminal globular domain shared by both A-type lamins, with no apparent effect on prelamin A processing [23]. The fact that not only pre-lamin A accumulation but also mutations affecting both A-type lamins can result in progeroid disease points to common molecular mechanisms underlying both, lamin A- (sporadic) and lamin A and C-related (hereditary) HGPS.

### Distinct morphological abnormalities in *LMNA^K542/K542N^* fibroblast nuclei

Comparison of the nuclear morphology revealed that the structural abnormalities of the nuclear lamina in *LMNA^K542/K542N^* fibroblasts substantially differs from those reported for sporadic HGPS [16], [17], [20]. In particular, similar to nuclei from the *LMNA^T528M/M540T^* patient [23], nuclei of our patients did not demonstrate multilobulation, but showed extensive protrusions frequently present at opposite poles, accompanied with honeycomb structures (Figure 1). Interestingly, similar to observations in the parents of the *LMNA^T528M/M540T^* patient [23], fibroblasts from the three healthy heterozygous K542N carriers showed similar nuclear protrusions, however, at a considerably lower frequency compared to the homozygous patients (5% vs. 28%). *LMNA^K542/K542N^* nuclei displayed partial mislocalization of emerin in most of the protrusions with a honeycomb pattern which hasn't been observed in *LMNA^G608G/+^* fibroblasts [18], [46]. Likewise, loss of lamin B1 within nuclear blebs, observed in our patients and the T528M/M540T patient, does not represent a typical nuclear abnormality in sporadic HGPS [20]. A reduction of the cellular amount of lamin B proteins, described in *LMNA^G608G/+^* cells, could not be confirmed by Western blotting in *LMNA^K542/K542N^* fibroblasts due to due to shortage of biological material. Microarray analysis, however, showed no transcriptional changes of LMNB1 and LMNB2, encoding lamin B1 and B2. In contrast to sporadic HGPS, K542N does not lead to LAP2 loss, but to its striking accumulation at the frontal edges of the nuclear protrusions, even though lamin A is frequently lost in these regions. This supports our previous assumption that K542N mutation impairs the association of the LAP2-lamin A complexes [4], [25], [47].

Our analysis of lamin A in skin and liver tissue provides direct evidence that the K542N mutation results in aberrant nuclear morphology *in vivo*. Thus far, *in vivo* effects of the G608G *LMNA* mutation were only indirectly assessed in a mouse line expressing human progerin in the epidermis [48]. In these mice keratinocyte nuclei had severe defects, including envelope lobulation and decreased nuclear circularity. Despite these defects, however, transgenic mice did not develop any of the skin abnormalities typical for HGPS. In contrast to the histopathological skin alterations in our patient, the hepatic nuclear abnormalities were not associated with any overt liver disease phenotype.

### Transcriptional signatures in *LMNA^K542/K542N^* fibroblasts

Comparison of the global gene expression profile in primary cultured skin fibroblasts from affected *LMNA^K542/K542N^*, healthy *LMNA^K542/+^* carriers and controls revealed the deregulation of 657 genes involved in developmental and cell differentiation pathways. *LMNA^K542/K542N^* and *LMNA^G608G/+^* fibroblasts showed substantial overlap in transcriptional signatures (13.7%; 90/657) [28], [29]. Among these were several genes, whose mutations caused human and/or mouse disorders that are either part of the HGPS phenotypic spectrum or affect tissues and/or organs which are also affected in HGPS. Seventy-five genes with a common transcriptional signature (83.3%) displayed concordant (matching) and 15 (16.7%) discordant (opposite) transcription patterns.

Among the differentially expressed genes specifically up-regulated in hereditary HGPS was the Wnt5a ligand, a member of the non-canonical Wnt signaling pathway, whose pathogenic role *in vivo* could be evidenced by increased expression in skin from an affected *LMNA^K542/K542N^* carrier. Wnt5a signaling is essential for normal developmental morphogenesis resulting in abnormal craniofacial and skeletal development in *Wnt5a* null mice [49], [50], and Robinow Syndrome in humans [51]. Moreover, Wnt5a was shown to be an important player in the decision-making process whether bone marrow mesenchymal cells differentiate into adipocytes or osteoblasts [45]. Since altered bone and fat metabolism belong to the major features of HGPS and other *LMNA*-associated progeroid disorders, the role of WNT5A in disease pathophysiology clearly warrants further investigations with the prospect to identify novel therapeutic avenues.

### Concordant transcriptional alterations in lamin A- and lamin A/C-related HGPS

Among the genes down-regulated in both HGPS types was *TWIST2*, a known transcription factor important for osteoblast differentiation and member of the NF-κB signalling pathway, whose knock-out (*Twist2^−/−^*) mouse model on a 129 genetic background displays a cachectic phenotype similar to HGPS [31], [32]. These mice are normal at birth, but thereafter develop severe wasting accompanied by adipose deficiency, skin atrophy with hyperkeratosis, absence/reduction of hair follicles and resulting in perinatal death from cachexia. On a 129/C57 mixed background, *Twist2^−/−^* mice develop a milder phenotype, similar to that of Setleis syndrome patients [32], [52], who develop focal facial dermal dysplasia, characterized by focal loss of subcutaneous fat and dermal appendages. Recently, homozygous nonsense mutations in *TWIST2* were described in patients with Setleis syndrome confirming that inactivation of *TWIST2* leads to a similar phenotype in humans [52]. Gene expression studies in sporadic and hereditary HGPS revealed a 3-fold down-regulation of *TWIST2* in cultured fibroblasts [28]. Immunohistochemical assessment of TWIST2 expression in skin from a homozygous K542N carrier provided *in vivo* evidence for its role in the pathogenesis of HGPS, with TWIST2 expression being lost in fibroblasts and periadenexial cells of the dermis. In mice, *Twist2* is expressed in the dermis as well as in the dermal sheath of mature hair follicles. The extensive phenotypic overlap between HGPS and Setleis syndrome patients as well as *Twist2^−/−^* mice, provides further evidence for a pivotal role of *TWIST2* in HGPS skin disease (Table 2).

**Table 2:** Phenotypic overlap between HGPS, Setleis syndrome patients, and Twist2*^−/−^* mice.

	HGPS patients	Setleis syndrome patients [52]	*Twist2^−/−^* 129/C57 mice [52]	*Twist2^−/−^* 129 mice [32]
**Skin changes**	generalized	focal	focal	generalized
**Subcutaneous fat**	reduced	near absence	absent	prominently reduced
**Hair**	alopecia	sparse hair	alopecia	sparse hair
**Hair follicles**	absent	n.m.	absent	reduced
**Sebaceous glands**	absent	n.m	absent	n.m.
**Sweat glands**	present	n.m.	absent	n.m.
**Eyelashes**	absent	absent lower eyelashes	absent lower eyelashes	n.m.

*Note:* n.m. denotes “not mentioned”.

### Discordant transcriptional alterations in lamin A- and lamin A/C-related HGPS

Comparison of the transcriptional signatures between *LMNA^K542/K542N^* and *LMNA^G608G/+^* fibroblasts revealed 15 (16.7%) genes with opposite changes in gene expression, among two known regulators of vascular calcification and bone metabolism, *ENPP1* and *OPG (TNFRSF11B)* [28]. Intriguingly, cardiovascular as well as bone disease represent major discordant phenotypic features in sporadic and hereditary HGPS. Whereas the latter is not associated with cardiovascular disease but exhibits severe focal osteolysis, sporadic HGPS is characterized by progressive cardiovascular disease with profound systemic adventitial fibrosis, frequent stenosis and calcification, as well as generalized features of atherosclerosis [38], [53], [54]. Unlike patients homozygous for K542N who die of respiratory failure due to severe pneumonia, the major cause of death in sporadic HGPS is myocardial infarction and stroke.

ENPP1 is a cell surface enzyme generating inorganic pyrophosphate (PPi), an essential physiologic inhibitor of calcification. Mutations decreasing *ENPP1* activity are associated with idiopathic infantile arterial calcification (GACI) as well as autosomal-recessive hypophosphatemic rickets [35], [55], [56]. Considering that a 30% decrease in *ENPP1* activity was already reported to cause GACI, the observed 6.8-fold decrease in *ENPP1* expression in *LMNA^G608G/+^* is expected to substantially contribute to vascular calcification whereas the 2.4-fold increase in *LMNA^K542/K542N^* fibroblasts may actually be protective with regard to vascular disease [28].

*OPG* is a member of the *OPG/RANK/RANKL* cytokine triad and a major secreted inhibitor of osteoclast maturation and activity [57], [58]. Increased OPG levels have been associated with the incidence and prevalence of vascular calcification and hence cardiovascular disease [33], [34], as well as with inactivating mutations in a hereditary bone disorder, autosomal recessive Juvenile Paget disease [59], [60], [61]. Accordingly, increased expression of *OPG* in sporadic HGPS is expected to have an impact on vascular calcification and disease, and, conversely, decreased expression in hereditary HGPS on bone disease. Thus the opposite expression patterns in *ENPP1* and *OPG* provide plausible molecular explanations for major phenotypic differences in sporadic and hereditary HGPS and point to common, but divergent underlying pathophysiological processes [4], [62]. Clearly, the extent of the contribution of *ENPP1* and *OPG* to the development of cardiovascular and bone disease needs to be further assessed. If confirmed, HGPS patients with decreased levels of OPG may actually profit from the treatment with recombinant osteoprotegerin which was shown to decrease bone resorption in patients with Juvenile Paget disease [63].

The different expression profiles regarding bone metabolism in sporadic and hereditary HGPS are likely to reflect differences in glucose metabolism. In contrast to patients with sporadic disease, who were shown in some cases to manifest insulin resistance or diabetes [5], [64], our patients displayed increased insulin sensitivity accompanied with elevated serum osteocalcin levels. C-peptide measurements in the elder affected sibs were repeatedly found to be very low, accompanied with decreased fasting and postprandial insulin levels during oGTT. Intriguingly, our findings point to a possible heterozygote effect since all *LMNA* K542N heterozygous family members, including the 16 year old heterozygous sister of the patients, showed moderately increased insulin sensitivity and reduced insulin response of the β–cell to glucose uptake, which led to impaired glucose tolerance/diabetes in the parents. Therefore, increased insulin sensitivity in the homozygous patients may actually be protective with regard to the consequences (diabetes) of reduced β–cell function. Furthermore, the fact that *LMNA* mutations can cause both, severe insulin resistance [65], [66], [67] and increased insulin sensitivity in target tissues, convincingly illustrates the pivotal role of A-type lamins in the regulation of glucose metabolism. Clearly, the possibly age related reduction of c-peptide levels observed in *LMNA* K542N homozygotes needs to be confirmed in larger studies.

**Figure 3:**
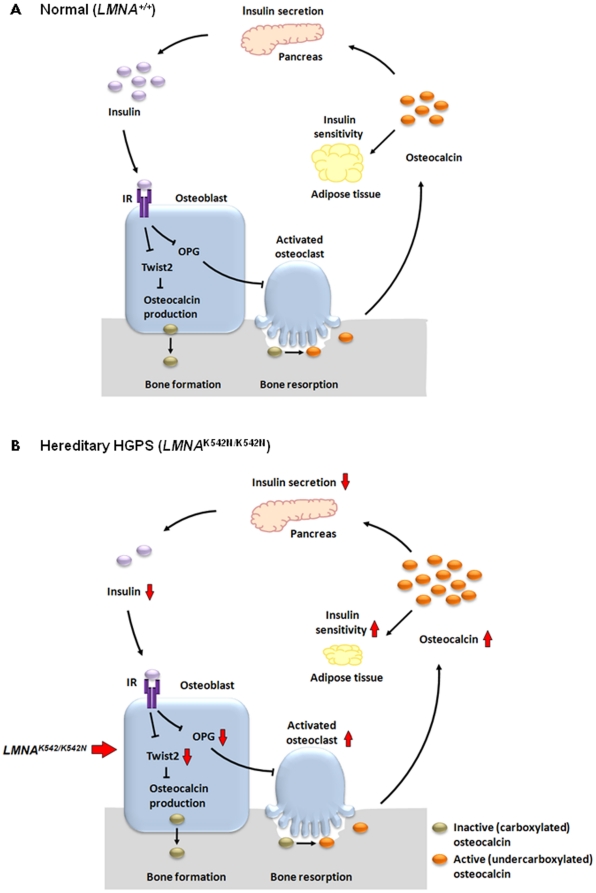
Effects of the *LMNA* K542N mutation on bone remodeling and energy metabolism through the insulin/osteocalcin axis. (A) Energy regulation and bone turnover by insulin signaling, adapted from Rosen and Motyl [74]. Insulin binds to the insulin receptor (IR) and activates bone remodeling by increasing bone formation by osteoblasts (through down-regulation of Twist2) and resorption by osteoclasts (though down-regulatin of OPG). Bone formation is coupled with the production of inactive (carboxylated) osteocalcin which is then released in an active form (undercarboxylated) during bone resorption and enters into the circulation. The active, hormonal form of osteocalcin enhances insulin secretion and increases the insulin sensitivity of adipocytes. (B) Altered regulation of energy metabolism and bone turnover in hereditary HGPS. In a homozygous state, the *LMNA* K542N mutation leads to the down-regulation of *TWIST2* and subsequent increased production of inactive osteocalcin. Decreased expression of *OPG* in *LMNA* K542N homozygotes enhances bone resorption, which then increases the release of the active, hormonal form of osteocalcin, and consequently results in increased insulin sensitivity. In a heterozygous state, the *LMNA* K542N mutation might overrule increased insulin sensitivity.

Previous discoveries in mice and human showed that osteocalcin, an osteoblast specific protein, acts as a hormone that increases insulin production, insulin sensitivity, and regulates fat deposition [44], [68], [69], [70], [71]. Intriguingly, very recent findings of two independent laboratories provide support that the complete bone remodeling process is involved in regulating energy metabolism, thus linking skeletal homeostasis to energy regulation [72], [73]. According to these findings, insulin signaling in osteoblasts mediates glucose homeostasis by stimulating the production of an inactive carboxylated form of osteocalcin and through down-regulation of *OPG* promotes osteoclast mediated bone resorption, which then releases the active undercarboxylated form of osteocalcin (Figure 3A; [74]). Interestingly, Fulzele et al. provided *in vivo* evidence that insulin signaling promotes bone formation by suppressing an inhibitor of osteoblast development, *Twist2*, and enhances expression of osteocalcin [72]. In line with this, the increased levels of osteocalcin detected in our patients could be due to down-regulation of *TWIST2*, with increased insulin sensitivity caused by increased bone resorption promoted by down-regulation of *OPG* (Figure 3B; [73]). Unfortunately, due to shortage of serum we could not assess in our patients the levels of serum OPG, undercarboxylated osteocalcin, as well as adiponectin, a known adipocyte specific cytokine regulated by osteocalcin and influencing insulin sensitivity [75].

Our findings raise many questions regarding energy metabolism not only in HGPS but, in view of recent discoveries in the field, also in humans in general. With the active form of osteocalcin being known as a potent stimulator of insulin secretion, the seemingly contradictory observations of increased osteocalcin levels and low levels of C-peptide and insulin observed in *LMNA* K542N homozygotes suggest the presence of other factors regulating insulin synthesis. Since insulin also has an impact on bone metabolism (Figure 3B, [44], [72], [73], [76], [77], [78]), it remains to be assessed whether decreased insulin production in K542N homozygotes directly contributes to bone disease. Because the liver is an important organ for energy balance and glucose metabolism [79], it remains to be seen if *in vivo* loss of TWIST2, observed in hepatocyte nuclei, affects insulin sensitivity/resistance and energy balance in hereditary HGPS. Do increased osteocalcin levels contribute to lipodystrophy given that its active (uncarboxylated) form is inversely correlated with fat deposition in mice and humans [71], [73], [80]? Since TWIST2 down-regulation has been observed in both HGPS types, inactive (carboxylated) osteocalcin levels are likely to be elevated in sporadic HGPS, too. Given the importance of osteocalcin in bone mineralization [81], do different ratios of active/inactive osteocalcin help to explain the differences in bone disease observed in sporadic and hereditary HGPS? Considering the reciprocal regulation of bone and energy metabolism, in which adipose tissue also controls bone remodeling [82], it remains to be answered which of the initial HGPS-related disease manifestations (failure to thrive, lipodystrophy, and bone disease [2], [4], [5], [62]) represent primary and which merely secondary consequences?

Altogether, our data indicate that the most typical symptoms of HGPS, such as bone disease, lipodystrophy, altered glucose metabolism, and cardiovascular disease are intimately linked and may be a consequence of altered skeletal and energy homeostasis. Clearly, our findings need to be confirmed in larger studies in order to further confirm the link between altered bone remodeling and energy homeostasis in hereditary HGPS. Our extensive comparative gene expression study corroborated by related clinical investigations has delineated several molecular processes altered in both of lamin A- and lamin A/C-related HGPS. Among the genes down-regulated in both types of HGPS is *TWIST2*, a known inhibitor of osteoblast development and member of the NF-κB signalling pathway, whose inactivation in mice and humans results in loss of subcutaneous fat and dermal appendages. Therefore, TWIST2 loss detected in fibroblasts and periadenexial cells of the dermis from a homozygous *LMNA* K542N patient provides direct *in vivo* evidence for its role in HGPS skin pathogenesis. Our observations of opposite transcriptional profiles in two key mediators of bone metabolism and vascular calcification, *ENPP1* and *OPG*, provide an explanation for divergent disease expression in sporadic and hereditary HGPS, i.e. the extent of vascular and bone disease. Further, this study correlates reduced *TWIST2* and *OPG* expression with increased osteocalcin levels and, consequently, insulin sensitivity, thereby linking altered bone remodeling to energy homeostasis in hereditary HGPS. Thus, this study recapitulates several recent findings on mouse models for bone remodeling and energy metabolism and exemplifies the value of hereditary disorders in delineating the mechanisms regulating energy homeostasis in humans.

## Materials and Methods

### Material

The clinical and *LMNA* mutation data on the autosomal recessive HGPS family has been previously reported [4]. In this study, we examined primary skin fibroblasts from affected homozygous (n = 3), healthy heterozygote carriers (n = 3), and healthy controls (n = 3), as well as autoptic skin and liver biopsies from a deceased family member. In addition, detailed clinical and laboratory follow-up of the family was undertaken.

### Ethics Statement

This study was approved by the ethics board of S.B. Devi Charity Home, Kolkata, India, and written informed consent was obtained from all family members.

### mRNA analysis

Total RNA was isolated from the primary skin fibroblasts (passage 2; 60-70% confluency) of affected homozygous K542N carriers (n = 3), healthy heterozygotes (n = 3), and wild-type controls (n = 3) using RNeasy Mini Kit (QIAGEN, Switzerland). Cells were lysed directly on the 100 mm culture plates using Buffer RLT and subsequently homogenized using QIAshredder spin columns (QIAGEN, Switzerland). RNA was extracted according to the manufacturer's protocol, quantified on NanoDrop 3300 (Thermo Scientific, Switzerland), and quality assessed on RNA Nano 6000 Chips (2100 Bioanalyzer, Agilent).

The K542N mRNA splicing was analyzed by reverse transcriptase (RT)-PCR using following primers: forward primer LMNA-ex8-12-F was located in exon 8 (5′-ACTGGAGTCCACTGAGAGCC-3′) and reverse primer LMNA-ex8-12-R in exon 12 (5′-GGCATGAGGTGAGGAGGAC-3′). RT-PCR and PCR were performed by means of QIAGEN OneStep RT-PCR Kit (QIAGEN, Switzerland) according to the manufacturer's protocol.

### Western analysis

Whole cells (2×10^6^) were collected, washed in PBS, and the pellets were resuspended in RIPA buffer containing a Halt Protease Inhibitor Cocktails (Thermo Scientific, Switzerland). Protein content was measured using BCA (bicinchoninic acid) protein assay (Thermo Scientific, Switzerland). Twenty micrograms of protein were loaded and electrophoresed on an 8% Tris-Glycine mini gel (Invitrogen, Switzerland). Proteins were then transferred onto a nitrocellulose membrane (Amersham Biosciences, Switzerlend), blocked with bovine serum albumin (3%), and incubated with anti-lamin A (133A2; ab8980, Abcam), anti-lamin A/C (JOL5, Acris Antibodies), and anti-actin (ab3280; Abcam) antibody as a loading control. After several washings, membranes were incubated with peroxidase-conjugated antibody (Jackson ImmunoResearch Laboratories, West Grove, PA), and immunoblots visualized by SuperSignal (Thermo Scientific, Switzerland).

### Immunofluorescence and nuclear morphometric analysis

To determine whether the K542N mutation leads to defects in nuclear architecture, immunofluorescence analysis of primary cultured skin fibroblasts (passages 2–5) was performed, using the following antibodies: anti-lamin A (133A2; ab8980, Abcam, and ab26300, Abcam), anti-lamin A/C (JOL5, Acris Antibodies), anti-LAP2 (27/LAP2, 611000, BD Biosciences, Milian, Switzerland), and anti-emerin (ab14208, Abcam). Shortly, cells were fixed 20 minutes in 2% PFA, permealized with 0.05% Triton X-100 for 5 minutes, and stained 1 hour with primary antibodies, followed by Alexa fluor 488 and 594 (Invitrogen), and mounted in ProLong Gold anti-fade reagent with DAPI (Invitrogen, Switzerland). Cells were observed on a Zeiss Axioskop optical microscope and Zeiss LSM 510 Meta confocal microscope (Zeiss, Germany). The overall percentage of malformed nuclei was scored in 200 nuclei, in a double-blind count. Because nuclei of K542N carriers manifested mostly single extensive lobe with typical ragged shape, the nuclei were scored as lobulated if they contained at least one such lobe. Statistical analyses were performed using unpaired, two-tailed Student's t test.

### Gene Expression profiling

Total RNA isolated from the primary skin fibroblasts (see above) was subjected to synthesis of double-stranded cDNA and biotin-labelled cRNA using GeneChip Expression 3′ Amplification One-Cycle Target labeling and Control reagents according to the manufacturer's protocol (Affymetrix). Fragmented cRNA preparations were hybridized to GeneChip® Human Genome U133 Plus 2.0 arrays (Affymetrix UK Ltd.) and scanned on a GeneChip Scanner 3000 7G (Affymetrix). All experiments were performed at the core facility, Life Sciences Training Facility, Division of Molecular Psychology, Biozentrum, and Department of Biomedicine, University of Basel. Microarray data is compliant to the minimum information about a microarray experiment (MIAME) criteria and is deposited at the European Bioinformatics Institute's ArrayExpress (www.ebi.ac.uk/arrayexpress; accession number E-MEXP-3097).

Affymetrix GeneChip CEL data files were processed and normalized using Robust Multi-Array (RMA) analysis and differential expression assessed by empirical Bayesian approach, as previously described [83]. P-value of <0.05 and fold change of 1.5 were used as a criteria to select the statistically significant differentially expressed genes. In addition, Ingenuity Pathways Analysis (Ingenuity Systems, www.ingenuity.com) software was used for interpretation of the results.

### Real-time quantitative PCR

Validation of the selected differentially expressed genes was performed using TaqMan Gene Expression Assays (Table S2; Applied Biosystems, Switzerland), according to the manufacturer's protocol. Actin, beta (ACTB) was used as an endogenous control and all reactions performed in triplicates. Relative gene expression changes were calculated using using the 2^−ΔΔCT^ method [84].

### Pathological examinations

Immunohistochemical evaluation of LMNA, TWIST2, and WNT5A was performed in skin and/or liver autoptic specimens from a deceased K542N homozygous patient according to the protocol previously described [85], using following antibodies: anti-lamin A (ab26300, Abcam), anti-Twist2 (3C8, M01, Abnova Corporation), and anti-Wnt5a (AF645, R&D Systems). Periodic acid-Schiff (PAS) staining for glycogen was performed using the standard procedures [86].

### Laboratory tests

The follow-up examination of insulin, C-peptide, glucose and lipids was performed in three patients, and healthy parents and sister and analyzed in the medical diagnostic laboratory Viollier AG (Switzerland). In a second, independent investigation, oral glucose tolerance testing was performed in two patients (A: male, age of 10, body weight (BW) 12 kg; E: male, age of 20, BW 13 kg) as well as the healthy parents (mother: age of 42, BW 60 kg; father: age of 47, BW 55 kg). After 12 hours of fasting the patients and their parents receiving 1.75 g of glucose per kilogram of body weight (maximum 75 g). Serum glucose and insulin were measured in Drs. Tribedi & Roy Diagnostic Laboratory (Kolkata, India) at 0 and 2 hours. Assessment of C-peptide and osteocalcin was performed at the medical diagnostic laboratory Viollier AG, Switzerland, and of TNF-alpha at the University Hospital, Zuerich. To assess insulin resistance, β-cell function and insulin sensitivity a nonlinear homeostatic model assessment (HOMA2) was undertaken [40]. In addition, indices derived from the oGTT were used to evaluate insulin sensitivity [42], [43]. Electrocardiogram and echocardiographic investigations were performed at the Institute of Child Health, Kolkata, and computed tomography (CT) imaging of the heart and the large vessels was performed at EKO X-Ray and Imaging Institute, Kolkata, India.

## Supporting Information

Figure S1 **RT-PCR analysis of** ***LMNA*** **mRNA in HGPS family** **carrying the LMNA K542N mutation.**
(DOC)

Figure S2 **Western blot analysis of A-type lamins in** ***LMNA^K542/K542N^*** **and** ***LMNA^K542/+^*** **fibroblasts.**
(DOC)

Table S1 **Gene expression signature in fibroblasts from patients homozygous for** ***LMNA K542N*** **.**
(DOC)

Table S2 **Validation of the microarray results with TaqMan Gene Expression Assays.**
(DOC)

Table S3 **Common gene expression signatures of the** ***LMNA*** **K542N and G608G mutation.** (A) Genes with concordant (matching) transcriptional patterns. (B) Genes with discordant (opposite) transcriptional patterns.
(DOC)

Table S4 **Overlap between** ***LMNA*** **G608G transcription signatures.**
(DOC)

Table S5 **Results of laboratory tests in the HGPS family carrying the** ***LMNA*** **K542N mutation.** (A) Laboratory tests. (B) Oral glucose tolerance test (oGTT)
(DOC)
