# Supplementary material for: Discordant Gene Expression Signatures and Related Phenotypic Differences in Lamin A- and A/C-Related Hutchinson-Gilford Progeria Syndrome (HGPS)
Source: PLoS One. 2011 Jun 27;6(6):e21433. doi: 10.1371/journal.pone.0021433 (PMC3124505; doi:10.1371/journal.pone.0021433)

**Figure S1. RT-PCR analysis of *LMNA* mRNA in HGPS family** **carrying the LMNA K542N mutation.**

RT-PCR analysis of *LMNA* mRNA from hetero- (empty symbols with dot) and homozygous (filled symbols) *LMNA* K542N mutation carriers indicates normal splicing of *LMNA*. Expected normal PCR product size: 797 bp, K: LMNA wild-type control, M: size marker


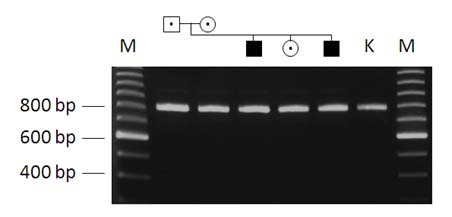

Supplement: Figure S1 — RT-PCR analysis of LMNA mRNA in HGPS family carrying the LMNA K542N mutation. (DOC) [file pone.0021433.s001.doc]
