# Supplementary material for: Discordant Gene Expression Signatures and Related Phenotypic Differences in Lamin A- and A/C-Related Hutchinson-Gilford Progeria Syndrome (HGPS)
Source: PLoS One. 2011 Jun 27;6(6):e21433. doi: 10.1371/journal.pone.0021433 (PMC3124505; doi:10.1371/journal.pone.0021433)
Supplement: Figure S2 — Western blot analysis of A-type lamins in LMNAK542/K542N and LMNAK542/+ fibroblasts. (DOC) [file pone.0021433.s002.doc]

**Figure S2. Western blot analysis of A-type lamins in *LMNA^K542/K542N^* and *LMNA^K542/+^* fibroblasts.**

Immunoblotting for lamin A and lamin A and C from hetero- (empty symbols with dot) and homozygous (filled symbols) *LMNA* K542N mutation carriers indicating similar expression patterns of A-type lamins. K: LMNA wild-type control, 133A2: lamin A specific antibody, JOL5: lamin A and C specific antibody.

**
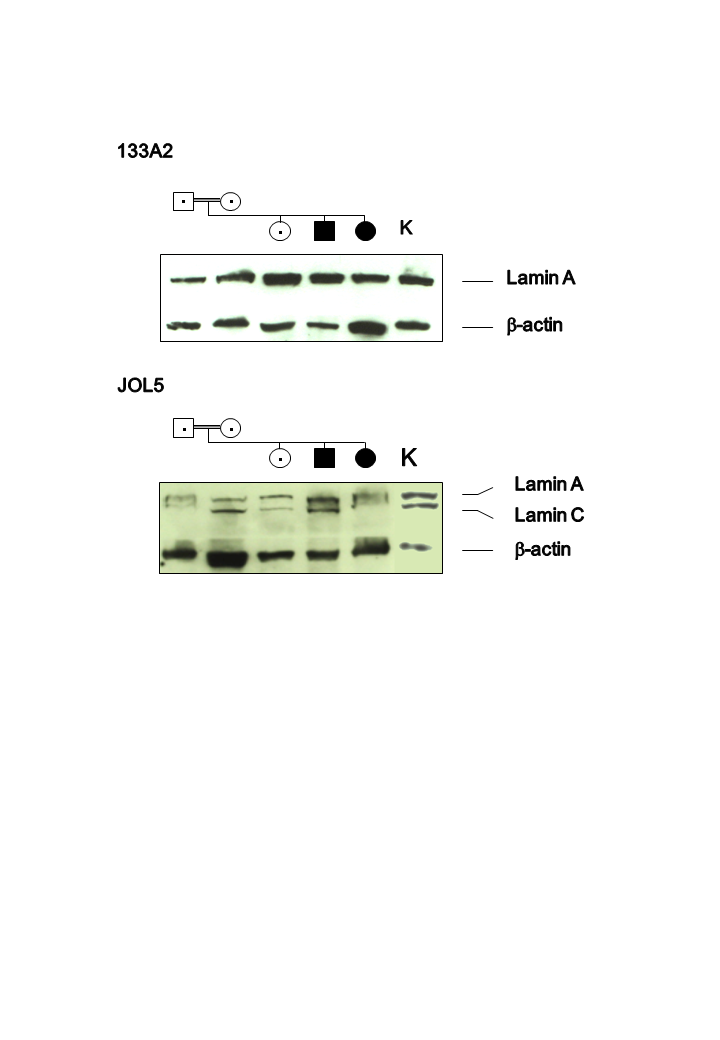
**

**
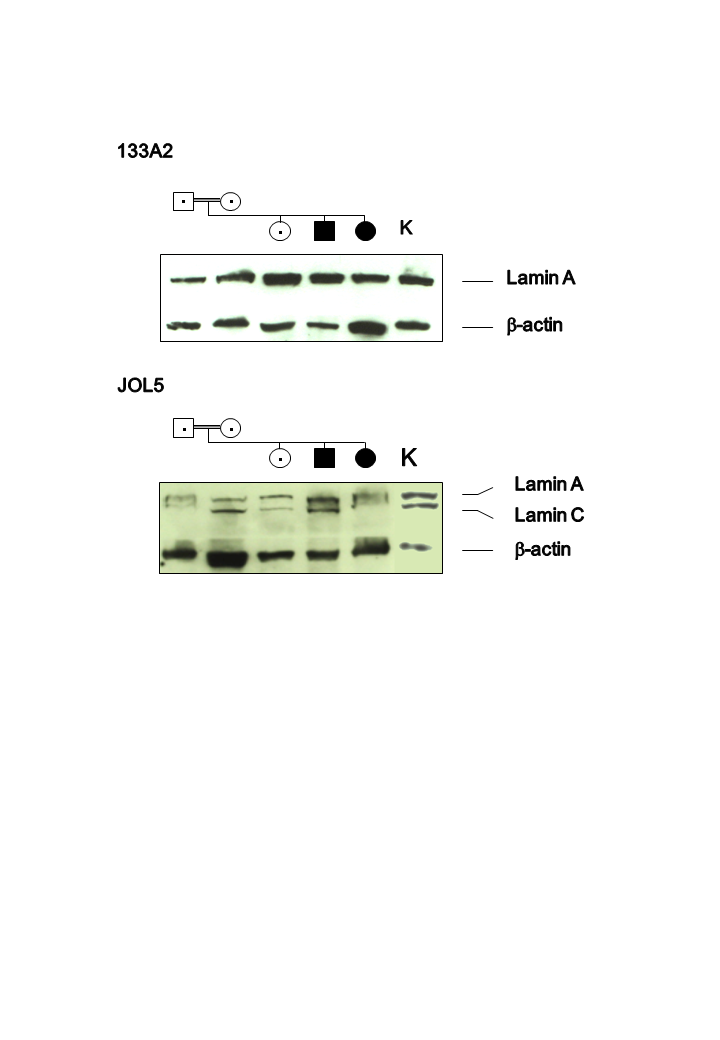
**
