# Supplementary material for: Discordant Gene Expression Signatures and Related Phenotypic Differences in Lamin A- and A/C-Related Hutchinson-Gilford Progeria Syndrome (HGPS)
Source: PLoS One. 2011 Jun 27;6(6):e21433. doi: 10.1371/journal.pone.0021433 (PMC3124505; doi:10.1371/journal.pone.0021433)
Supplement: Table S1 — Gene expression signature in fibroblasts from patients homozygous for LMNA K542N . (DOC) [file pone.0021433.s003.doc]

**Table S1. Gene expression signature in fibroblasts from patients homozygous for LMNA K542N.**

| **Probe Set ID** | **Gene Symbol** | **Gene Name** | **Location** | **Type** | **A** | **FC** | **p-value** |
| --- | --- | --- | --- | --- | --- | --- | --- |
| 205792_at | WISP2 | WNT1 inducible signaling pathway protein 2 | Extracellular Space | growth factor | 9.34 | -7.06 | 4.57E-03 |
| 205110_s_at | FGF13 | fibroblast growth factor 13 | Extracellular Space | growth factor | 8.35 | -6.45 | 5.74E-05 |
| 227949_at | PHACTR3 | phosphatase and actin regulator 3 | Nucleus | other | 5.88 | -5.82 | 5.64E-05 |
| 213241_at | PLXNC1 | plexin C1 | Plasma Membrane | other | 8.57 | -5.66 | 3.08E-03 |
| 228716_at | THRB | thyroid hormone receptor, beta (erythroblastic leukemia viral (v-erb-a) oncogene homolog 2, avian) | Nucleus | ligand-dependent nuclear receptor | 6.67 | -5.62 | 4.14E-04 |
| 229116_at | CNKSR2 | connector enhancer of kinase suppressor of Ras 2 | Plasma Membrane | other | 5.85 | -4.66 | 6.37E-04 |
| 208396_s_at | PDE1A | phosphodiesterase 1A, calmodulin-dependent | Cytoplasm | enzyme | 5.65 | -4.56 | 1.18E-02 |
| 213415_at | CLIC2 | chloride intracellular channel 2 | unknown | other | 5.63 | -4.53 | 2.30E-03 |
| 208131_s_at | PTGIS | prostaglandin I2 (prostacyclin) synthase | Cytoplasm | enzyme | 10.91 | -4.41 | 1.53E-03 |
| 228186_s_at | RSPO3 | R-spondin 3 homolog (Xenopus laevis) | unknown | kinase | 8.76 | -4.35 | 6.11E-03 |
| 218918_at | MAN1C1 | mannosidase, alpha, class 1C, member 1 | Cytoplasm | enzyme | 6.34 | -4.08 | 6.69E-03 |
| 207145_at | MSTN | myostatin | Extracellular Space | growth factor | 4.68 | -4.08 | 6.60E-03 |
| 232267_at | GPR133 | G protein-coupled receptor 133 | Plasma Membrane | G-protein coupled receptor | 7.71 | -4.03 | 2.03E-02 |
| 219304_s_at | PDGFD | platelet derived growth factor D | Extracellular Space | growth factor | 8.05 | -4.00 | 3.73E-02 |
| 230360_at | GLDN | gliomedin | Cytoplasm | other | 5.91 | -3.94 | 1.23E-02 |
| 201693_s_at | EGR1 | early growth response 1 | Nucleus | transcription regulator | 7.52 | -3.66 | 3.75E-02 |
| 203180_at | ALDH1A3 | aldehyde dehydrogenase 1 family, member A3 | Cytoplasm | enzyme | 12.22 | -3.53 | 1.23E-03 |
| 239461_at | GALNTL2 | UDP-N-acetyl-alpha-D-galactosamine:polypeptide N-acetylgalactosaminyltransferase-like 2 | Cytoplasm | enzyme | 6.99 | -3.53 | 8.43E-03 |
| 1554163_at | TWIST2 | twist homolog 2 (Drosophila) | Nucleus | transcription regulator | 8.46 | -3.46 | 1.14E-04 |
| 241399_at | FAM19A2 | family with sequence similarity 19 (chemokine (C-C motif)-like), member A2 | Cytoplasm | other | 6.13 | -3.43 | 5.19E-03 |
| 1559005_s_at | CNTLN | centlein, centrosomal protein | unknown | other | 5.09 | -3.41 | 2.07E-05 |
| 211653_x_at | AKR1C2 | aldo-keto reductase family 1, member C2 (dihydrodiol dehydrogenase 2 | Cytoplasm | enzyme | 10.64 | -3.39 | 2.10E-06 |
| 213068_at | DPT | dermatopontin | Extracellular Space | other | 6.43 | -3.36 | 6.47E-03 |
| 204151_x_at | AKR1C1 | aldo-keto reductase family 1, member C1 (dihydrodiol dehydrogenase 1; 20-alpha (3-alpha)-hydroxysteroid dehydrogenase) | Cytoplasm | enzyme | 11.53 | -3.34 | 1.07E-06 |
| 217220_at | DKFZP586K1520 | DKFZP586K1520 protein | unknown | other | 7.97 | -3.29 | 6.87E-04 |
| 209160_at | AKR1C3 | aldo-keto reductase family 1, member C3 (3-alpha hydroxysteroid dehydrogenase, type II) | Cytoplasm | enzyme | 9.77 | -3.29 | 3.11E-04 |
| 206025_s_at | TNFAIP6 | tumor necrosis factor, alpha-induced protein 6 | Extracellular Space | other | 8.73 | -3.29 | 3.45E-02 |
| 218692_at | SYBU | syntabulin (syntaxin-interacting) | unknown | other | 6.55 | -3.25 | 1.88E-03 |
| 205382_s_at | CFD | complement factor D (adipsin) | Extracellular Space | peptidase | 6.63 | -3.07 | 9.77E-04 |
| 226926_at | DMKN | dermokine | unknown | other | 5.80 | -3.05 | 1.23E-03 |
| 220351_at | CCRL1 | chemokine (C-C motif) receptor-like 1 | Plasma Membrane | G-protein coupled receptor | 8.56 | -3.03 | 2.83E-02 |
| 212390_at | PDE4DIP | phosphodiesterase 4D interacting protein | Cytoplasm | enzyme | 9.25 | -3.03 | 3.42E-03 |
| 226145_s_at | FRAS1 | Fraser syndrome 1 | Extracellular Space | other | 9.26 | -2.91 | 2.93E-03 |
| 225016_at | APCDD1 | adenomatosis polyposis coli down-regulated 1 | unknown | other | 7.73 | -2.89 | 1.46E-02 |
| 230137_at | TMEM155 | transmembrane protein 155 | unknown | other | 8.06 | -2.85 | 6.24E-04 |
| 209596_at | MXRA5 | matrix-remodelling associated 5 | unknown | other | 10.70 | -2.77 | 5.56E-04 |
| 1555097_a_at | PTGFR | prostaglandin F receptor (FP) | Plasma Membrane | G-protein coupled receptor | 7.00 | -2.77 | 2.81E-03 |
| 204040_at | RNF144A | ring finger protein 144A | Nucleus | other | 6.65 | -2.73 | 9.48E-05 |
| 201787_at | FBLN1 | fibulin 1 | Extracellular Space | other | 8.94 | -2.73 | 1.66E-02 |
| 213125_at | OLFML2B | olfactomedin-like 2B | unknown | other | 8.03 | -2.73 | 1.33E-05 |
| 215184_at | DAPK2 | death-associated protein kinase 2 | Cytoplasm | kinase | 6.94 | -2.69 | 6.37E-03 |
| 222101_s_at | DCHS1 | dachsous 1 (Drosophila) | Plasma Membrane | other | 7.64 | -2.69 | 1.23E-03 |
| 222895_s_at | BCL11B | B-cell CLL/lymphoma 11B (zinc finger protein) | Nucleus | other | 4.40 | -2.64 | 1.28E-02 |
| 228707_at | CLDN23 | claudin 23 | Plasma Membrane | other | 4.42 | -2.62 | 5.24E-03 |
| 228262_at | MAP7D2 | MAP7 domain containing 2 | unknown | other | 6.56 | -2.62 | 2.30E-04 |
| 219454_at | EGFL6 | EGF-like-domain, multiple 6 | Extracellular Space | other | 4.96 | -2.60 | 4.11E-02 |
| 206363_at | MAF | v-maf musculoaponeurotic fibrosarcoma oncogene homolog (avian) | Nucleus | transcription regulator | 5.91 | -2.60 | 4.39E-03 |
| 206805_at | SEMA3A | sema domain, immunoglobulin domain (Ig), short basic domain, secreted, (semaphorin) 3A | Extracellular Space | other | 8.84 | -2.58 | 2.63E-05 |
| 230925_at | APBB1IP | amyloid beta (A4) precursor protein-binding, family B, member 1 interacting protein | Cytoplasm | other | 9.57 | -2.57 | 4.57E-04 |
| 228665_at | CYYR1 | cysteine/tyrosine-rich 1 | unknown | other | 5.36 | -2.57 | 7.51E-03 |
| 225990_at | BOC | Boc homolog (mouse) | unknown | other | 8.87 | -2.55 | 7.53E-04 |
| 213790_at | ADAM12 | ADAM metallopeptidase domain 12 | Plasma Membrane | peptidase | 8.54 | -2.53 | 1.50E-02 |
| 211478_s_at | DPP4 | dipeptidyl-peptidase 4 | Plasma Membrane | peptidase | 6.24 | -2.51 | 2.00E-02 |
| 216250_s_at | LPXN | leupaxin | Cytoplasm | other | 10.81 | -2.51 | 2.86E-04 |
| 204368_at | SLCO2A1 | solute carrier organic anion transporter family, member 2A1 | Plasma Membrane | transporter | 6.34 | -2.51 | 2.37E-03 |
| 201792_at | AEBP1 | AE binding protein 1 | Nucleus | peptidase | 9.60 | -2.48 | 8.44E-04 |
| 230087_at | PRIMA1 | proline rich membrane anchor 1 | unknown | other | 4.72 | -2.46 | 1.41E-02 |
| 221019_s_at | COLEC12 | collectin sub-family member 12 | Plasma Membrane | transmembrane receptor | 11.07 | -2.45 | 1.27E-02 |
| 204639_at | ADA | adenosine deaminase | Cytoplasm | enzyme | 9.36 | -2.39 | 3.03E-03 |
| 215116_s_at | DNM1 | dynamin 1 | Cytoplasm | enzyme | 8.07 | -2.39 | 3.81E-05 |
| 228575_at | IL20RB | interleukin 20 receptor beta | Plasma Membrane | other | 5.60 | -2.38 | 3.27E-02 |
| 238636_at | CACNA1C | calcium channel, voltage-dependent, L type, alpha 1C subunit | Plasma Membrane | ion channel | 5.73 | -2.36 | 2.90E-03 |
| 203666_at | CXCL12 | chemokine (C-X-C motif) ligand 12 | Extracellular Space | cytokine | 10.83 | -2.36 | 2.03E-05 |
| 212946_at | KIAA0564 | KIAA0564 | unknown | other | 7.95 | -2.36 | 1.82E-02 |
| 208070_s_at | REV3L | REV3-like, catalytic subunit of DNA polymerase zeta (yeast) | Nucleus | enzyme | 9.40 | -2.35 | 1.68E-02 |
| 213001_at | ANGPTL2 | angiopoietin-like 2 | Extracellular Space | other | 8.96 | -2.31 | 2.97E-02 |
| 227250_at | KREMEN1 | kringle containing transmembrane protein 1 | Plasma Membrane | other | 6.69 | -2.28 | 5.55E-03 |
| 1557570_a_at | LOC285084 | hypothetical protein LOC285084 | unknown | other | 4.01 | -2.28 | 4.15E-02 |
| 223168_at | RHOU | ras homolog gene family, member U | Cytoplasm | enzyme | 5.91 | -2.28 | 1.82E-02 |
| 203435_s_at | MME | membrane metallo-endopeptidase | Plasma Membrane | peptidase | 10.44 | -2.27 | 3.92E-02 |
| 204933_s_at | TNFRSF11B | tumor necrosis factor receptor superfamily, member 11b | Plasma Membrane | transmembrane receptor | 10.53 | -2.27 | 2.32E-02 |
| 229357_at | ADAMTS5 | ADAM metallopeptidase with thrombospondin type 1 motif, 5 | Extracellular Space | peptidase | 9.44 | -2.27 | 1.79E-02 |
| 37170_at | BMP2K | BMP2 inducible kinase | Nucleus | kinase | 5.17 | -2.27 | 2.73E-03 |
| 229437_at | MIR155HG | MIR155 host gene (non-protein coding) | unknown | other | 6.36 | -2.27 | 9.04E-04 |
| 225056_at | SIPA1L2 | signal-induced proliferation-associated 1 like 2 | unknown | other | 5.60 | -2.27 | 2.58E-02 |
| 220117_at | ZNF385D | zinc finger protein 385D | Nucleus | other | 5.55 | -2.27 | 1.95E-06 |
| 207057_at | SLC16A7 | solute carrier family 16, member 7 (monocarboxylic acid transporter 2) | Plasma Membrane | transporter | 8.88 | -2.25 | 5.07E-05 |
| 212148_at | PBX1 | pre-B-cell leukemia homeobox 1 | Nucleus | transcription regulator | 9.79 | -2.23 | 5.87E-04 |
| 202357_s_at | CFB | complement factor B | Extracellular Space | peptidase | 6.02 | -2.23 | 8.64E-03 |
| 205578_at | ROR2 | receptor tyrosine kinase-like orphan receptor 2 | Plasma Membrane | kinase | 5.24 | -2.20 | 2.13E-04 |
| 227791_at | SLC9A9 | solute carrier family 9 (sodium/hydrogen exchanger), member 9 | Cytoplasm | other | 7.41 | -2.20 | 2.77E-02 |
| 230472_at | IRX1 | iroquois homeobox 1 | Nucleus | transcription regulator | 8.63 | -2.19 | 4.92E-04 |
| 229172_at | HSPA12B | heat shock 70kD protein 12B | unknown | other | 5.09 | -2.16 | 2.74E-03 |
| 226925_at | ACPL2 | acid phosphatase-like 2 | unknown | phosphatase | 7.72 | -2.14 | 6.09E-06 |
| 229831_at | CNTN3 | contactin 3 (plasmacytoma associated) | Plasma Membrane | other | 10.00 | -2.14 | 2.85E-05 |
| 229943_at | TRIM13 | tripartite motif-containing 13 | Cytoplasm | other | 7.29 | -2.14 | 3.13E-02 |
| 201427_s_at | SEPP1 | selenoprotein P, plasma, 1 | Extracellular Space | other | 10.42 | -2.13 | 2.31E-02 |
| 208022_s_at | CDC14B | CDC14 cell division cycle 14 homolog B (S. cerevisiae) | Nucleus | phosphatase | 7.80 | -2.13 | 5.08E-03 |
| 225627_s_at | CACHD1 | cache domain containing 1 | unknown | other | 8.14 | -2.11 | 6.15E-04 |
| 205403_at | IL1R2 | interleukin 1 receptor, type II | Plasma Membrane | transmembrane receptor | 5.90 | -2.11 | 3.62E-02 |
| 203475_at | CYP19A1 | cytochrome P450, family 19, subfamily A, polypeptide 1 | Cytoplasm | enzyme | 5.27 | -2.10 | 1.18E-02 |
| 204580_at | MMP12 | matrix metallopeptidase 12 (macrophage elastase) | Extracellular Space | peptidase | 8.82 | -2.10 | 3.76E-02 |
| 225871_at | STEAP2 | six transmembrane epithelial antigen of the prostate 2 | Plasma Membrane | transporter | 8.83 | -2.10 | 2.36E-03 |
| 202350_s_at | MATN2 | matrilin 2 | Extracellular Space | other | 9.37 | -2.08 | 2.06E-03 |
| 222773_s_at | GALNT12 | UDP-N-acetyl-alpha-D-galactosamine:polypeptide N-acetylgalactosaminyltransferase 12 (GalNAc-T12) | Cytoplasm | enzyme | 6.05 | -2.07 | 2.10E-02 |
| 206375_s_at | HSPB3 | heat shock 27kDa protein 3 | unknown | other | 7.73 | -2.07 | 3.66E-02 |
| 1559315_s_at | LOC144481 | hypothetical protein LOC144481 | unknown | other | 5.41 | -2.07 | 3.94E-02 |
| 243940_at | TSHZ2 | teashirt zinc finger homeobox 2 | unknown | other | 4.93 | -2.07 | 2.65E-02 |
| 1552274_at | PXK | PX domain containing serine/threonine kinase | Cytoplasm | kinase | 8.40 | -2.06 | 1.64E-03 |
| 218559_s_at | MAFB | v-maf musculoaponeurotic fibrosarcoma oncogene homolog B (avian) | Nucleus | other | 10.36 | -2.04 | 1.18E-02 |
| 218002_s_at | CXCL14 | chemokine (C-X-C motif) ligand 14 | Extracellular Space | cytokine | 5.92 | -2.04 | 1.22E-02 |
| 212850_s_at | LRP4 | low density lipoprotein receptor-related protein 4 | Extracellular Space | other | 7.59 | -2.04 | 3.24E-04 |
| 238332_at | ANKRD29 | ankyrin repeat domain 29 | unknown | other | 6.39 | -2.03 | 3.32E-02 |
| 223710_at | CCL26 | chemokine (C-C motif) ligand 26 | Extracellular Space | cytokine | 6.78 | -2.03 | 4.69E-05 |
| 205031_at | EFNB3 | ephrin-B3 | Plasma Membrane | kinase | 6.30 | -2.03 | 2.98E-03 |
| 242907_at | GBP2 | guanylate binding protein 2, interferon-inducible | Cytoplasm | enzyme | 6.19 | -2.01 | 4.99E-02 |
| 227910_at | XPNPEP3 | X-prolyl aminopeptidase (aminopeptidase P) 3, putative | unknown | peptidase | 6.42 | -2.01 | 2.17E-02 |
| 1559901_s_at | C21orf34 | chromosome 21 open reading frame 34 | unknown | other | 3.84 | -1.99 | 8.66E-03 |
| 228302_x_at | CAMK2N1 | calcium/calmodulin-dependent protein kinase II inhibitor 1 | Plasma Membrane | kinase | 6.22 | -1.99 | 4.76E-03 |
| 238621_at | FMN1 | formin 1 | Nucleus | transporter | 5.92 | -1.99 | 6.49E-03 |
| 203498_at | RCAN2 | regulator of calcineurin 2 | unknown | other | 8.65 | -1.99 | 4.57E-02 |
| 223126_s_at | C1orf21 | chromosome 1 open reading frame 21 | unknown | other | 7.74 | -1.97 | 1.99E-03 |
| 53991_at | DENND2A | DENN/MADD domain containing 2A | unknown | other | 6.14 | -1.97 | 1.10E-02 |
| 203088_at | FBLN5 | fibulin 5 | Extracellular Space | other | 11.34 | -1.97 | 1.79E-02 |
| 210139_s_at | PMP22 | peripheral myelin protein 22 | Plasma Membrane | other | 11.45 | -1.97 | 1.17E-02 |
| 213935_at | ABHD5 | abhydrolase domain containing 5 | Cytoplasm | enzyme | 7.06 | -1.96 | 1.28E-04 |
| 231729_s_at | CAPS | calcyphosine | Cytoplasm | other | 5.33 | -1.96 | 3.37E-04 |
| 220102_at | FOXL2 | forkhead box L2 | Nucleus | transcription regulator | 5.82 | -1.96 | 3.95E-02 |
| 206606_at | LIPC | lipase, hepatic | Extracellular Space | enzyme | 5.31 | -1.96 | 1.22E-02 |
| 235272_at | SBSN | suprabasin | unknown | other | 7.25 | -1.96 | 4.64E-03 |
| 206403_at | ZNF536 | zinc finger protein 536 | unknown | other | 6.61 | -1.96 | 2.70E-02 |
| 205003_at | DOCK4 | dedicator of cytokinesis 4 | Plasma Membrane | other | 5.21 | -1.95 | 5.66E-03 |
| 227678_at | XRCC6BP1 | XRCC6 binding protein 1 | unknown | kinase | 6.05 | -1.95 | 4.08E-04 |
| 52837_at | KIAA1644 | KIAA1644 | unknown | other | 7.25 | -1.93 | 2.92E-02 |
| 226071_at | ADAMTSL4 | ADAMTS-like 4 | unknown | other | 5.59 | -1.93 | 4.22E-02 |
| 205696_s_at | GFRA1 | GDNF family receptor alpha 1 | Plasma Membrane | transmembrane receptor | 5.82 | -1.93 | 3.33E-02 |
| 204678_s_at | KCNK1 | potassium channel, subfamily K, member 1 | Plasma Membrane | ion channel | 6.62 | -1.93 | 9.10E-03 |
| 205794_s_at | NOVA1 | neuro-oncological ventral antigen 1 | Nucleus | other | 6.39 | -1.93 | 1.53E-02 |
| 202975_s_at | RHOBTB3 | Rho-related BTB domain containing 3 | unknown | enzyme | 9.86 | -1.93 | 2.16E-02 |
| 213285_at | TMEM30B | transmembrane protein 30B | unknown | other | 6.25 | -1.93 | 3.52E-02 |
| 224374_s_at | EMILIN2 | elastin microfibril interfacer 2 | Extracellular Space | other | 9.36 | -1.92 | 2.88E-02 |
| 208965_s_at | IFI16 | interferon, gamma-inducible protein 16 | Nucleus | transcription regulator | 9.26 | -1.92 | 2.58E-02 |
| 202948_at | IL1R1 | interleukin 1 receptor, type I | Plasma Membrane | transmembrane receptor | 11.43 | -1.92 | 1.91E-02 |
| 202450_s_at | CTSK | cathepsin K | Cytoplasm | peptidase | 11.88 | -1.91 | 7.16E-03 |
| 207505_at | PRKG2 | protein kinase, cGMP-dependent, type II | Cytoplasm | kinase | 6.47 | -1.91 | 5.10E-04 |
| 210839_s_at | ENPP2 | ectonucleotide pyrophosphatase/phosphodiesterase 2 | Plasma Membrane | enzyme | 12.35 | -1.89 | 7.77E-03 |
| 207808_s_at | PROS1 | protein S (alpha) | Extracellular Space | other | 8.88 | -1.89 | 2.91E-02 |
| 205021_s_at | FOXN3 | forkhead box N3 | Nucleus | transcription regulator | 6.90 | -1.89 | 2.85E-03 |
| 205968_at | KCNS3 | potassium voltage-gated channel, delayed-rectifier, subfamily S, member 3 | Plasma Membrane | ion channel | 6.46 | -1.88 | 4.60E-03 |
| 211368_s_at | CASP1 | caspase 1, apoptosis-related cysteine peptidase (interleukin 1, beta, convertase) | Cytoplasm | peptidase | 6.26 | -1.88 | 2.51E-02 |
| 205627_at | CDA | cytidine deaminase | Nucleus | enzyme | 6.87 | -1.88 | 1.67E-03 |
| 218532_s_at | FAM134B | family with sequence similarity 134, member B | unknown | other | 4.98 | -1.88 | 3.25E-03 |
| 203886_s_at | FBLN2 | fibulin 2 | Extracellular Space | other | 9.76 | -1.88 | 3.87E-02 |
| 230619_at | ARNT | aryl hydrocarbon receptor nuclear translocator | Nucleus | transcription regulator | 7.38 | -1.87 | 6.55E-04 |
| 236649_at | DTWD1 | DTW domain containing 1 | unknown | other | 7.57 | -1.87 | 1.68E-02 |
| 223611_s_at | LNX1 | ligand of numb-protein X 1 | Cytoplasm | enzyme | 7.63 | -1.87 | 1.37E-02 |
| 219525_at | SLC47A1 | solute carrier family 47, member 1 | unknown | other | 5.33 | -1.85 | 5.80E-03 |
| 226606_s_at | GTPBP5 | GTP binding protein 5 (putative) | unknown | other | 6.98 | -1.84 | 4.16E-02 |
| 227067_x_at | NOTCH2NL | Notch homolog 2 (Drosophila) N-terminal like | unknown | other | 9.12 | -1.84 | 7.21E-03 |
| 205542_at | STEAP1 | six transmembrane epithelial antigen of the prostate 1 | Plasma Membrane | transporter | 11.47 | -1.83 | 2.13E-04 |
| 230963_at | EMX2OS | EMX2 opposite strand (non-protein coding) | unknown | other | 8.83 | -1.83 | 4.16E-03 |
| 209147_s_at | PPAP2A | phosphatidic acid phosphatase type 2A | Plasma Membrane | phosphatase | 10.65 | -1.83 | 9.20E-03 |
| 209307_at | SWAP70 | SWAP switching B-cell complex 70kDa subunit | Cytoplasm | other | 9.51 | -1.83 | 7.19E-03 |
| 209897_s_at | SLIT2 | slit homolog 2 (Drosophila) | Extracellular Space | other | 10.99 | -1.82 | 4.68E-04 |
| 227223_at | RBM39 | RNA binding motif protein 39 | Nucleus | transcription regulator | 6.86 | -1.79 | 2.69E-02 |
| 202283_at | SERPINF1 | serpin peptidase inhibitor, clade F (alpha-2 antiplasmin, pigment epithelium derived factor), member 1 | Extracellular Space | other | 9.57 | -1.79 | 1.31E-02 |
| 238430_x_at | SLFN5 | schlafen family member 5 | Nucleus | enzyme | 8.44 | -1.79 | 1.68E-03 |
| 223730_at | GPC6 | glypican 6 | Plasma Membrane | transmembrane receptor | 7.71 | -1.78 | 4.67E-02 |
| 221814_at | GPR124 | G protein-coupled receptor 124 | Plasma Membrane | G-protein coupled receptor | 8.62 | -1.78 | 2.37E-02 |
| 224497_x_at | HSD17B14 | hydroxysteroid (17-beta) dehydrogenase 14 | unknown | enzyme | 6.17 | -1.78 | 1.01E-02 |
| 202732_at | PKIG | protein kinase (cAMP-dependent, catalytic) inhibitor gamma | unknown | other | 10.62 | -1.78 | 3.77E-02 |
| 227660_at | ANTXR1 | anthrax toxin receptor 1 | Plasma Membrane | other | 10.53 | -1.77 | 1.65E-04 |
| 202156_s_at | CELF2 | CUGBP, Elav-like family member 2 | Nucleus | other | 7.40 | -1.77 | 1.22E-02 |
| 228250_at | FNIP1 | folliculin interacting protein 1 | unknown | other | 6.76 | -1.77 | 2.84E-04 |
| 203628_at | IGF1R | insulin-like growth factor 1 receptor | Plasma Membrane | transmembrane receptor | 7.65 | -1.77 | 2.11E-02 |
| 204334_at | KLF7 | Kruppel-like factor 7 (ubiquitous) | Nucleus | transcription regulator | 8.96 | -1.77 | 3.17E-02 |
| 202446_s_at | PLSCR1 | phospholipid scramblase 1 | Plasma Membrane | enzyme | 9.14 | -1.77 | 1.11E-02 |
| 244689_at | PPARA | peroxisome proliferator-activated receptor alpha | Nucleus | ligand-dependent nuclear receptor | 6.22 | -1.77 | 1.32E-02 |
| 230000_at | RNF213 | ring finger protein 213 | Plasma Membrane | other | 5.92 | -1.77 | 1.21E-02 |
| 227072_at | RTTN | rotatin | unknown | other | 8.38 | -1.77 | 2.99E-03 |
| 212257_s_at | SMARCA2 | SWI/SNF related, matrix associated, actin dependent regulator of chromatin, subfamily a, member 2 | Nucleus | transcription regulator | 7.01 | -1.77 | 4.63E-03 |
| 229851_s_at | C11orf54 | chromosome 11 open reading frame 54 | Nucleus | other | 7.10 | -1.75 | 8.67E-03 |
| 203910_at | ARHGAP29 | Rho GTPase activating protein 29 | Cytoplasm | other | 10.63 | -1.75 | 9.39E-03 |
| 212240_s_at | PIK3R1 | phosphoinositide-3-kinase, regulatory subunit 1 (alpha) | Cytoplasm | kinase | 8.73 | -1.75 | 3.83E-04 |
| 201852_x_at | COL3A1 | collagen, type III, alpha 1 | Extracellular Space | other | 13.01 | -1.74 | 1.63E-02 |
| 218326_s_at | LGR4 | leucine-rich repeat-containing G protein-coupled receptor 4 | Plasma Membrane | G-protein coupled receptor | 8.76 | -1.74 | 1.18E-02 |
| 224218_s_at | TRPS1 | trichorhinophalangeal syndrome I | Nucleus | transcription regulator | 6.49 | -1.74 | 8.48E-03 |
| 216985_s_at | STX3 | syntaxin 3 | Plasma Membrane | transporter | 4.95 | -1.73 | 1.82E-03 |
| 204301_at | KBTBD11 | kelch repeat and BTB (POZ) domain containing 11 | unknown | other | 5.62 | -1.72 | 2.47E-02 |
| 209785_s_at | PLA2G4C | phospholipase A2, group IVC (cytosolic, calcium-independent) | Cytoplasm | enzyme | 5.61 | -1.72 | 1.12E-02 |
| 219684_at | RTP4 | receptor (chemosensory) transporter protein 4 | Plasma Membrane | other | 5.35 | -1.72 | 2.74E-02 |
| 211896_s_at | DCN | decorin | Extracellular Space | other | 12.88 | -1.71 | 3.10E-02 |
| 213645_at | ENOSF1 | enolase superfamily member 1 | unknown | other | 6.77 | -1.71 | 1.19E-03 |
| 1554966_a_at | FILIP1L | filamin A interacting protein 1-like | Nucleus | other | 9.87 | -1.71 | 7.26E-04 |
| 228462_at | IRX2 | iroquois homeobox 2 | Nucleus | transcription regulator | 9.21 | -1.71 | 6.20E-04 |
| 203372_s_at | SOCS2 | suppressor of cytokine signaling 2 | Cytoplasm | other | 8.49 | -1.71 | 3.84E-02 |
| 234973_at | SLC38A5 | solute carrier family 38, member 5 | unknown | transporter | 7.21 | -1.69 | 1.21E-02 |
| 210380_s_at | CACNA1G | calcium channel, voltage-dependent, T type, alpha 1G subunit | Plasma Membrane | ion channel | 5.79 | -1.69 | 1.54E-02 |
| 206371_at | FOLR3 | folate receptor 3 (gamma) | Extracellular Space | other | 6.62 | -1.69 | 1.28E-03 |
| 207110_at | KCNJ12 | potassium inwardly-rectifying channel, subfamily J, member 12 | Plasma Membrane | ion channel | 4.44 | -1.69 | 8.24E-04 |
| 239093_at | DHDPSL | dihydrodipicolinate synthase-like, mitochondrial | Cytoplasm | other | 5.05 | -1.68 | 1.69E-02 |
| 204773_at | IL11RA | interleukin 11 receptor, alpha | Plasma Membrane | transmembrane receptor | 8.67 | -1.68 | 4.46E-02 |
| 220783_at | MMP27 | matrix metallopeptidase 27 | unknown | peptidase | 4.22 | -1.68 | 5.03E-03 |
| 205529_s_at | RUNX1T1 | runt-related transcription factor 1; translocated to, 1 (cyclin D-related) | Nucleus | transcription regulator | 8.52 | -1.68 | 1.32E-02 |
| 224631_at | ZFP91 | zinc finger protein 91 homolog (mouse) | Nucleus | transcription regulator | 6.80 | -1.68 | 3.36E-04 |
| 228667_at | AGPAT4 | 1-acylglycerol-3-phosphate O-acyltransferase 4 (lysophosphatidic acid acyltransferase, delta) | Cytoplasm | enzyme | 6.45 | -1.67 | 1.46E-02 |
| 202760_s_at | AKAP2 /// PALM2-AKAP2 | A kinase (PRKA) anchor protein 2 /// PALM2-AKAP2 readthrough | Cytoplasm | other | 11.19 | -1.67 | 2.66E-03 |
| 206449_s_at | MASP1 | mannan-binding lectin serine peptidase 1 (C4/C2 activating component of Ra-reactive factor) | Extracellular Space | peptidase | 4.98 | -1.67 | 1.94E-02 |
| 232138_at | MBNL2 | Muscleblind-like 2 (Drosophila) | unknown | other | 5.89 | -1.67 | 2.39E-02 |
| 218330_s_at | NAV2 | neuron navigator 2 | Nucleus | other | 9.25 | -1.67 | 4.90E-03 |
| 227478_at | SETBP1 | SET binding protein 1 | Nucleus | other | 6.55 | -1.67 | 2.85E-02 |
| 206377_at | FOXF2 | forkhead box F2 | Nucleus | transcription regulator | 8.42 | -1.66 | 2.35E-03 |
| 226334_s_at | AHSA2 | AHA1, activator of heat shock 90kDa protein ATPase homolog 2 (yeast) | unknown | other | 7.56 | -1.66 | 3.79E-02 |
| 221211_s_at | C21orf7 | chromosome 21 open reading frame 7 | unknown | other | 7.73 | -1.66 | 5.44E-03 |
| 201743_at | CD14 | CD14 molecule | Plasma Membrane | transmembrane receptor | 5.60 | -1.66 | 3.20E-02 |
| 1564796_at | EMP1 | epithelial membrane protein 1 | Plasma Membrane | other | 6.55 | -1.66 | 2.77E-02 |
| 212677_s_at | CEP68 | centrosomal protein 68kDa | Cytoplasm | other | 6.68 | -1.65 | 2.10E-02 |
| 223657_at | FAM167B | family with sequence similarity 167, member B | unknown | other | 6.39 | -1.65 | 1.27E-02 |
| 201465_s_at | JUN | jun oncogene | Nucleus | transcription regulator | 7.61 | -1.65 | 2.17E-03 |
| 209750_at | NR1D2 | nuclear receptor subfamily 1, group D, member 2 | Nucleus | ligand-dependent nuclear receptor | 9.50 | -1.65 | 4.07E-02 |
| 222528_s_at | SLC25A37 | solute carrier family 25, member 37 | Cytoplasm | transporter | 6.66 | -1.65 | 1.63E-03 |
| 213820_s_at | STARD5 | StAR-related lipid transfer (START) domain containing 5 | Cytoplasm | transporter | 7.49 | -1.65 | 6.37E-04 |
| 205992_s_at | IL15 | interleukin 15 | Extracellular Space | cytokine | 7.54 | -1.64 | 1.96E-02 |
| 242324_x_at | CCBE1 | collagen and calcium binding EGF domains 1 | unknown | other | 8.57 | -1.62 | 4.76E-03 |
| 203096_s_at | RAPGEF2 | Rap guanine nucleotide exchange factor (GEF) 2 | Cytoplasm | other | 7.53 | -1.62 | 9.18E-04 |
| 223028_s_at | SNX9 | sorting nexin 9 | Cytoplasm | transporter | 10.00 | -1.62 | 5.54E-03 |
| 201286_at | SDC1 | syndecan 1 | Plasma Membrane | other | 9.59 | -1.61 | 5.77E-04 |
| 201625_s_at | INSIG1 | insulin induced gene 1 | Cytoplasm | other | 7.73 | -1.61 | 1.32E-03 |
| 230486_at | PCBP3 | poly(rC) binding protein 3 | Nucleus | other | 7.34 | -1.61 | 1.39E-02 |
| 206908_s_at | CLDN11 | claudin 11 | Plasma Membrane | other | 8.52 | -1.60 | 1.75E-02 |
| 204194_at | BACH1 | BTB and CNC homology 1, basic leucine zipper transcription factor 1 | Nucleus | transcription regulator | 7.95 | -1.60 | 4.58E-02 |
| 224458_at | C9orf125 | chromosome 9 open reading frame 125 | unknown | other | 7.47 | -1.60 | 1.83E-03 |
| 228790_at | FAM110B | family with sequence similarity 110, member B | unknown | other | 7.85 | -1.60 | 1.84E-02 |
| 1569290_s_at | GRIA3 | glutamate receptor, ionotrophic, AMPA 3 | Plasma Membrane | ion channel | 8.28 | -1.60 | 1.76E-02 |
| 228813_at | HDAC4 | histone deacetylase 4 | Nucleus | transcription regulator | 5.09 | -1.60 | 4.32E-02 |
| 202421_at | IGSF3 | immunoglobulin superfamily, member 3 | Plasma Membrane | other | 5.66 | -1.60 | 3.16E-03 |
| 222413_s_at | MLL3 | myeloid/lymphoid or mixed-lineage leukemia 3 | Nucleus | transcription regulator | 6.40 | -1.60 | 1.35E-03 |
| 204917_s_at | MLLT3 | myeloid/lymphoid or mixed-lineage leukemia (trithorax homolog, Drosophila); translocated to, 3 | Nucleus | other | 6.88 | -1.60 | 1.13E-02 |
| 228176_at | S1PR3 | sphingosine-1-phosphate receptor 3 | Plasma Membrane | G-protein coupled receptor | 9.29 | -1.60 | 1.74E-02 |
| 225564_at | SPATA13 | spermatogenesis associated 13 | unknown | other | 6.81 | -1.60 | 3.26E-02 |
| 226875_at | DOCK11 | dedicator of cytokinesis 11 | unknown | other | 8.50 | -1.59 | 2.45E-02 |
| 201431_s_at | DPYSL3 | dihydropyrimidinase-like 3 | Cytoplasm | enzyme | 8.95 | -1.59 | 8.77E-03 |
| 1555103_s_at | FGF7 | fibroblast growth factor 7 (keratinocyte growth factor) | Extracellular Space | growth factor | 6.08 | -1.59 | 4.76E-02 |
| 229625_at | GBP5 | guanylate binding protein 5 | Plasma Membrane | enzyme | 5.56 | -1.59 | 3.92E-05 |
| 207375_s_at | IL15RA | interleukin 15 receptor, alpha | Plasma Membrane | transmembrane receptor | 6.42 | -1.59 | 2.93E-02 |
| 227379_at | MBOAT1 | membrane bound O-acyltransferase domain containing 1 | unknown | other | 5.62 | -1.59 | 1.17E-02 |
| 202679_at | NPC1 | Niemann-Pick disease, type C1 | Cytoplasm | transporter | 9.50 | -1.59 | 2.30E-03 |
| 228964_at | PRDM1 | PR domain containing 1, with ZNF domain | Nucleus | transcription regulator | 7.24 | -1.59 | 8.74E-03 |
| 206027_at | S100A3 | S100 calcium binding protein A3 | unknown | transporter | 7.91 | -1.59 | 2.09E-03 |
| 205330_at | MN1 | meningioma (disrupted in balanced translocation) 1 | Nucleus | other | 9.17 | -1.58 | 3.08E-02 |
| 1555894_s_at | MTSS1L | metastasis suppressor 1-like | unknown | other | 6.32 | -1.58 | 2.10E-02 |
| 238041_at | TCF12 | transcription factor 12 | Nucleus | transcription regulator | 8.00 | -1.58 | 2.99E-04 |
| 202054_s_at | ALDH3A2 | aldehyde dehydrogenase 3 family, member A2 | Cytoplasm | enzyme | 7.82 | -1.57 | 1.25E-02 |
| 207387_s_at | GK | glycerol kinase | Cytoplasm | kinase | 5.85 | -1.57 | 8.67E-03 |
| 1556425_a_at | LOC284219 | hypothetical protein LOC284219 | unknown | other | 5.64 | -1.57 | 2.74E-02 |
| 214433_s_at | SELENBP1 | selenium binding protein 1 | Cytoplasm | other | 7.51 | -1.57 | 3.51E-02 |
| 213035_at | ANKRD28 | ankyrin repeat domain 28 | unknown | other | 8.29 | -1.56 | 2.01E-03 |
| 239612_at | LOC100240734 | hypothetical LOC100240734 | unknown | other | 4.56 | -1.56 | 7.27E-04 |
| 236154_at | QKI | quaking homolog, KH domain RNA binding (mouse) | Nucleus | other | 4.76 | -1.56 | 2.79E-02 |
| 221999_at | VRK3 | vaccinia related kinase 3 | unknown | kinase | 6.79 | -1.56 | 2.56E-02 |
| 210944_s_at | CAPN3 | calpain 3, (p94) | Cytoplasm | peptidase | 6.28 | -1.55 | 3.84E-03 |
| 206114_at | EPHA4 | EPH receptor A4 | Plasma Membrane | kinase | 5.84 | -1.55 | 2.97E-02 |
| 225511_at | GPRC5B | G protein-coupled receptor, family C, group 5, member B | Plasma Membrane | G-protein coupled receptor | 4.58 | -1.55 | 3.00E-02 |
| 205680_at | MMP10 | matrix metallopeptidase 10 (stromelysin 2) | Extracellular Space | peptidase | 4.93 | -1.55 | 2.80E-02 |
| 205203_at | PLD1 | phospholipase D1, phosphatidylcholine-specific | Cytoplasm | enzyme | 6.02 | -1.54 | 3.84E-02 |
| 215049_x_at | CD163 | CD163 molecule | Plasma Membrane | transmembrane receptor | 4.08 | -1.54 | 9.54E-03 |
| 207386_at | CYP7B1 | cytochrome P450, family 7, subfamily B, polypeptide 1 | Cytoplasm | enzyme | 3.49 | -1.54 | 4.27E-02 |
| 206980_s_at | FLT3LG | fms-related tyrosine kinase 3 ligand | Extracellular Space | cytokine | 5.83 | -1.54 | 2.59E-02 |
| 225955_at | METRNL | meteorin, glial cell differentiation regulator-like | unknown | other | 10.00 | -1.54 | 3.08E-03 |
| 47069_at | PRR5 | proline rich 5 (renal) | Cytoplasm | other | 7.33 | -1.54 | 4.41E-02 |
| 204140_at | TPST1 | tyrosylprotein sulfotransferase 1 | Cytoplasm | enzyme | 9.45 | -1.54 | 5.66E-03 |
| 212361_s_at | ATP2A2 | ATPase, Ca++ transporting, cardiac muscle, slow twitch 2 | Cytoplasm | transporter | 9.93 | -1.53 | 7.78E-03 |
| 203368_at | CRELD1 | cysteine-rich with EGF-like domains 1 | unknown | other | 6.63 | -1.53 | 2.62E-02 |
| 229295_at | LOC150166 | hypothetical protein LOC150166 | unknown | other | 7.14 | -1.53 | 6.33E-03 |
| 230412_at | NPAS3 | neuronal PAS domain protein 3 | Nucleus | other | 3.52 | -1.53 | 3.22E-02 |
| 202604_x_at | ADAM10 | ADAM metallopeptidase domain 10 | Plasma Membrane | peptidase | 8.97 | -1.52 | 3.37E-03 |
| 224414_s_at | CARD6 | caspase recruitment domain family, member 6 | Cytoplasm | other | 8.51 | -1.52 | 3.63E-02 |
| 203799_at | CD302 | CD302 molecule | Plasma Membrane | transmembrane receptor | 7.88 | -1.52 | 1.80E-02 |
| 202382_s_at | GNPDA1 | glucosamine-6-phosphate deaminase 1 | Cytoplasm | enzyme | 9.12 | -1.52 | 1.12E-02 |
| 226225_at | MCC | mutated in colorectal cancers | unknown | other | 7.10 | -1.52 | 4.69E-02 |
| 242935_at | SBF2 | SET binding factor 2 | Cytoplasm | other | 6.39 | -1.52 | 2.29E-02 |
| 214830_at | SLC38A6 | solute carrier family 38, member 6 | unknown | transporter | 8.64 | -1.52 | 3.86E-02 |
| 214919_s_at | ANKHD1-EIF4EBP3 /// EIF4EBP3 | ANKHD1-EIF4EBP3 readthrough /// eukaryotic translation initiation factor 4E binding protein 3 | unknown | other | 7.39 | -1.51 | 1.48E-02 |
| 219437_s_at | ANKRD11 | ankyrin repeat domain 11 | Nucleus | other | 6.16 | -1.51 | 4.07E-02 |
| 227534_at | C9orf21 | chromosome 9 open reading frame 21 | unknown | other | 9.05 | -1.51 | 2.49E-02 |
| 235721_at | DTX3 | deltex homolog 3 (Drosophila) | Cytoplasm | other | 7.22 | -1.51 | 3.20E-02 |
| 225602_at | GLIPR2 | GLI pathogenesis-related 2 | Cytoplasm | other | 9.50 | -1.51 | 7.72E-03 |
| 219269_at | HMBOX1 | homeobox containing 1 | Nucleus | transcription regulator | 6.97 | -1.51 | 4.10E-02 |
| 218486_at | KLF11 | Kruppel-like factor 11 | Nucleus | transcription regulator | 7.12 | -1.51 | 3.53E-02 |
| 218129_s_at | NFYB | nuclear transcription factor Y, beta | Nucleus | transcription regulator | 8.32 | -1.51 | 1.69E-02 |
| 204188_s_at | RARG | retinoic acid receptor, gamma | Nucleus | ligand-dependent nuclear receptor | 7.59 | -1.51 | 3.32E-03 |
| 228248_at | RICTOR | RPTOR independent companion of MTOR, complex 2 | Cytoplasm | other | 7.03 | -1.51 | 4.63E-02 |
| 215411_s_at | TRAF3IP2 | TRAF3 interacting protein 2 | unknown | other | 8.77 | -1.51 | 2.56E-02 |
| 208527_x_at | HIST1H2BE | histone cluster 1, H2be | Nucleus | other | 6.78 | 1.50 | 1.50E-03 |
| 202363_at | SPOCK1 | sparc/osteonectin, cwcv and kazal-like domains proteoglycan (testican) 1 | Extracellular Space | other | 10.27 | 1.50 | 1.25E-03 |
| 223501_at | TNFSF13B | tumor necrosis factor (ligand) superfamily, member 13b | Extracellular Space | cytokine | 4.59 | 1.50 | 3.20E-02 |
| 226611_s_at | CENPV | centromere protein V | Nucleus | other | 5.85 | 1.51 | 2.44E-03 |
| 222701_s_at | CHCHD7 | coiled-coil-helix-coiled-coil-helix domain containing 7 | unknown | other | 9.21 | 1.51 | 1.64E-03 |
| 220565_at | CCR10 | chemokine (C-C motif) receptor 10 | Plasma Membrane | G-protein coupled receptor | 4.79 | 1.51 | 1.23E-02 |
| 230903_s_at | C8orf42 | chromosome 8 open reading frame 42 | unknown | other | 4.26 | 1.51 | 4.92E-06 |
| 1556597_a_at | LOC284513 | hypothetical protein LOC284513 | unknown | other | 3.87 | 1.51 | 1.45E-04 |
| 212774_at | ZNF238 | zinc finger protein 238 | Nucleus | transcription regulator | 8.25 | 1.51 | 2.67E-03 |
| 207426_s_at | TNFSF4 | tumor necrosis factor (ligand) superfamily, member 4 | Extracellular Space | cytokine | 4.13 | 1.51 | 8.66E-03 |
| 207787_at | KRT33B | keratin 33B | Cytoplasm | other | 5.09 | 1.52 | 3.82E-02 |
| 206757_at | PDE5A | phosphodiesterase 5A, cGMP-specific | Cytoplasm | enzyme | 3.79 | 1.52 | 3.04E-02 |
| 209806_at | HIST1H2BK | histone cluster 1, H2bk | Nucleus | other | 10.08 | 1.52 | 2.96E-03 |
| 1554378_a_at | PDE1C | phosphodiesterase 1C, calmodulin-dependent 70kDa | Cytoplasm | enzyme | 3.58 | 1.52 | 7.07E-03 |
| 204993_at | GNAZ | guanine nucleotide binding protein (G protein), alpha z polypeptide | Plasma Membrane | enzyme | 5.81 | 1.52 | 1.12E-03 |
| 200730_s_at | PTP4A1 | protein tyrosine phosphatase type IVA, member 1 | Nucleus | phosphatase | 9.03 | 1.52 | 3.17E-03 |
| 227055_at | METTL7B | methyltransferase like 7B | unknown | enzyme | 5.31 | 1.53 | 1.81E-02 |
| 202388_at | RGS2 | regulator of G-protein signaling 2, 24kDa | Nucleus | other | 7.39 | 1.53 | 2.89E-02 |
| 209791_at | PADI2 | peptidyl arginine deiminase, type II | Cytoplasm | enzyme | 5.41 | 1.53 | 1.85E-03 |
| 205304_s_at | KCNJ8 | potassium inwardly-rectifying channel, subfamily J, member 8 | Plasma Membrane | ion channel | 6.62 | 1.53 | 1.97E-02 |
| 207528_s_at | SLC7A11 | solute carrier family 7, (cationic amino acid transporter, y+ system) member 11 | Plasma Membrane | transporter | 4.81 | 1.54 | 2.33E-02 |
| 204256_at | ELOVL6 | ELOVL family member 6, elongation of long chain fatty acids (FEN1/Elo2, SUR4/Elo3-like, yeast) | Cytoplasm | enzyme | 7.73 | 1.54 | 3.38E-02 |
| 203642_s_at | COBLL1 | COBL-like 1 | unknown | other | 6.51 | 1.54 | 1.68E-03 |
| 201438_at | COL6A3 | collagen, type VI, alpha 3 | Extracellular Space | other | 12.01 | 1.54 | 1.59E-02 |
| 210687_at | CPT1A | carnitine palmitoyltransferase 1A (liver) | Cytoplasm | enzyme | 4.91 | 1.54 | 7.68E-03 |
| 228490_at | ABHD2 | abhydrolase domain containing 2 | unknown | enzyme | 6.60 | 1.55 | 3.77E-02 |
| 208579_x_at | H2BFS | H2B histone family, member S | Nucleus | other | 8.82 | 1.55 | 3.00E-03 |
| 202998_s_at | LOXL2 | lysyl oxidase-like 2 | Extracellular Space | enzyme | 11.23 | 1.55 | 7.97E-03 |
| 202613_at | CTPS | CTP synthase | Nucleus | enzyme | 8.94 | 1.55 | 4.36E-02 |
| 233823_at | FAM184B | family with sequence similarity 184, member B | unknown | other | 4.92 | 1.55 | 7.11E-03 |
| 218975_at | COL5A3 | collagen, type V, alpha 3 | Extracellular Space | other | 6.00 | 1.55 | 1.36E-03 |
| 207012_at | MMP16 | matrix metallopeptidase 16 (membrane-inserted) | Extracellular Space | peptidase | 5.33 | 1.55 | 3.82E-02 |
| 218484_at | NDUFA4L2 | NADH dehydrogenase (ubiquinone) 1 alpha subcomplex, 4-like 2 | unknown | enzyme | 5.96 | 1.56 | 1.60E-03 |
| 228990_at | SNHG12 | small nucleolar RNA host gene 12 (non-protein coding) | unknown | other | 6.89 | 1.56 | 1.48E-02 |
| 230906_at | GALNT10 | UDP-N-acetyl-alpha-D-galactosamine:polypeptide N-acetylgalactosaminyltransferase 10 (GalNAc-T10) | Cytoplasm | enzyme | 7.21 | 1.56 | 8.91E-05 |
| 201579_at | FAT1 | FAT tumor suppressor homolog 1 (Drosophila) | Plasma Membrane | other | 9.13 | 1.56 | 8.37E-05 |
| 204115_at | GNG11 | guanine nucleotide binding protein (G protein), gamma 11 | Plasma Membrane | enzyme | 10.75 | 1.56 | 9.70E-03 |
| 226609_at | DCBLD1 | discoidin, CUB and LCCL domain containing 1 | Extracellular Space | other | 8.55 | 1.56 | 8.51E-03 |
| 230112_at | MARCH4 | membrane-associated ring finger (C3HC4) 4 | unknown | other | 8.32 | 1.57 | 3.93E-02 |
| 224838_at | FOXP1 | forkhead box P1 | Nucleus | transcription regulator | 9.23 | 1.57 | 3.34E-03 |
| 203157_s_at | GLS | glutaminase | Cytoplasm | enzyme | 8.89 | 1.57 | 3.57E-02 |
| 225314_at | OCIAD2 | OCIA domain containing 2 | unknown | other | 8.06 | 1.57 | 2.05E-02 |
| 212554_at | CAP2 | CAP, adenylate cyclase-associated protein, 2 (yeast) | Plasma Membrane | other | 8.68 | 1.57 | 2.49E-03 |
| 218796_at | FERMT1 | fermitin family homolog 1 (Drosophila) | Plasma Membrane | other | 6.28 | 1.57 | 9.27E-04 |
| 1554004_a_at | RGNEF | 190 kDa guanine nucleotide exchange factor | unknown | other | 4.92 | 1.58 | 3.41E-02 |
| 234772_s_at | KAP2.1B /// KRTAP2-2 | keratin associated protein 2.1B /// keratin associated protein 2-2 | unknown | other | 3.87 | 1.58 | 4.02E-04 |
| 203349_s_at | ETV5 | ets variant 5 | Nucleus | transcription regulator | 7.97 | 1.58 | 3.10E-02 |
| 1554195_a_at | C5orf46 | chromosome 5 open reading frame 46 | unknown | other | 4.49 | 1.58 | 7.73E-03 |
| 240770_at | TMEM171 | transmembrane protein 171 | unknown | other | 5.92 | 1.58 | 4.98E-03 |
| 230900_at | CCDC110 | coiled-coil domain containing 110 | unknown | other | 3.28 | 1.58 | 4.63E-03 |
| 205749_at | CYP1A1 | cytochrome P450, family 1, subfamily A, polypeptide 1 | Cytoplasm | enzyme | 4.30 | 1.58 | 4.33E-02 |
| 205450_at | PHKA1 | phosphorylase kinase, alpha 1 (muscle) | Cytoplasm | kinase | 5.26 | 1.58 | 6.23E-03 |
| 229830_at | PDGFA | Platelet-derived growth factor alpha polypeptide | Extracellular Space | growth factor | 5.71 | 1.58 | 1.46E-02 |
| 201079_at | SYNGR2 | synaptogyrin 2 | Plasma Membrane | other | 6.27 | 1.58 | 2.77E-05 |
| 226517_at | BCAT1 | branched chain amino-acid transaminase 1, cytosolic | Cytoplasm | enzyme | 10.28 | 1.59 | 3.82E-02 |
| 206814_at | NGF | nerve growth factor (beta polypeptide) | Extracellular Space | growth factor | 8.24 | 1.59 | 8.87E-04 |
| 242963_at | SGMS2 | sphingomyelin synthase 2 | Plasma Membrane | enzyme | 5.30 | 1.59 | 1.02E-03 |
| 200965_s_at | ABLIM1 | actin binding LIM protein 1 | Cytoplasm | other | 5.35 | 1.59 | 7.66E-03 |
| 213782_s_at | MYOZ2 | myozenin 2 | Cytoplasm | other | 4.38 | 1.59 | 1.40E-02 |
| 224818_at | SORT1 | sortilin 1 | Cytoplasm | transporter | 8.10 | 1.59 | 1.41E-02 |
| 201236_s_at | BTG2 | BTG family, member 2 | Nucleus | transcription regulator | 6.22 | 1.59 | 8.41E-03 |
| 241871_at | CAMK4 | calcium/calmodulin-dependent protein kinase IV | Nucleus | kinase | 3.29 | 1.59 | 1.65E-03 |
| 227401_at | IL17D | interleukin 17D | Extracellular Space | other | 4.63 | 1.60 | 3.28E-02 |
| 207076_s_at | ASS1 | argininosuccinate synthase 1 | Cytoplasm | enzyme | 9.67 | 1.60 | 4.04E-03 |
| 209946_at | VEGFC | vascular endothelial growth factor C | Extracellular Space | growth factor | 10.34 | 1.60 | 1.68E-02 |
| 212944_at | SLC5A3 | solute carrier family 5 (sodium/myo-inositol cotransporter), member 3 | Plasma Membrane | transporter | 8.08 | 1.60 | 4.59E-02 |
| 230643_at | WNT9A | wingless-type MMTV integration site family, member 9A | Extracellular Space | other | 5.54 | 1.60 | 2.47E-02 |
| 237737_at | LOC100289026 | similar to hCG1744891 | unknown | other | 3.58 | 1.61 | 8.73E-03 |
| 206440_at | LIN7A | lin-7 homolog A (C. elegans) | Cytoplasm | other | 4.53 | 1.61 | 7.06E-03 |
| 224823_at | MYLK | myosin light chain kinase | Cytoplasm | kinase | 10.79 | 1.61 | 1.26E-02 |
| 206197_at | NME5 | non-metastatic cells 5, protein expressed in (nucleoside-diphosphate kinase) | unknown | kinase | 5.29 | 1.61 | 1.04E-02 |
| 225589_at | SH3RF1 | SH3 domain containing ring finger 1 | Cytoplasm | other | 8.79 | 1.61 | 1.53E-03 |
| 223655_at | CD163L1 | CD163 molecule-like 1 | Plasma Membrane | transmembrane receptor | 5.50 | 1.61 | 1.30E-03 |
| 219298_at | ECHDC3 | enoyl CoA hydratase domain containing 3 | unknown | enzyme | 6.77 | 1.63 | 2.75E-02 |
| 223276_at | C5orf62 | chromosome 5 open reading frame 62 | unknown | ion channel | 9.20 | 1.63 | 5.03E-04 |
| 203999_at | SYT1 | synaptotagmin I | Cytoplasm | transporter | 4.72 | 1.63 | 4.47E-02 |
| 201809_s_at | ENG | endoglin | Plasma Membrane | other | 9.81 | 1.64 | 3.91E-04 |
| 219032_x_at | OPN3 | opsin 3 | Plasma Membrane | G-protein coupled receptor | 7.43 | 1.64 | 6.01E-03 |
| 231859_at | C14orf132 | chromosome 14 open reading frame 132 | unknown | other | 7.75 | 1.64 | 1.20E-02 |
| 215071_s_at | HIST1H2AC | histone cluster 1, H2ac | Nucleus | other | 8.11 | 1.64 | 2.64E-03 |
| 209949_at | NCF2 | neutrophil cytosolic factor 2 | Cytoplasm | enzyme | 4.33 | 1.64 | 2.37E-02 |
| 218888_s_at | NETO2 | neuropilin (NRP) and tolloid (TLL)-like 2 | unknown | other | 6.78 | 1.65 | 1.49E-02 |
| 201015_s_at | JUP | junction plakoglobin | Plasma Membrane | other | 5.95 | 1.65 | 1.50E-03 |
| 237460_x_at | C14orf182 | chromosome 14 open reading frame 182 | unknown | other | 4.79 | 1.65 | 1.97E-03 |
| 237032_x_at | LOC283567 | hypothetical protein LOC283567 | unknown | other | 5.52 | 1.65 | 1.74E-03 |
| 217744_s_at | PERP | PERP, TP53 apoptosis effector | Plasma Membrane | other | 9.18 | 1.65 | 1.94E-03 |
| 204005_s_at | PAWR | PRKC, apoptosis, WT1, regulator | Nucleus | transcription regulator | 8.15 | 1.65 | 1.44E-02 |
| 203185_at | RASSF2 | Ras association (RalGDS/AF-6) domain family member 2 | Nucleus | other | 5.53 | 1.65 | 1.13E-02 |
| 201690_s_at | TPD52 | tumor protein D52 | Cytoplasm | other | 2.89 | 1.65 | 1.06E-02 |
| 202193_at | LIMK2 | LIM domain kinase 2 | Cytoplasm | kinase | 7.50 | 1.65 | 8.94E-03 |
| 243409_at | FOXL1 | forkhead box L1 | Nucleus | transcription regulator | 7.12 | 1.66 | 1.05E-02 |
| 201445_at | CNN3 | calponin 3, acidic | Cytoplasm | other | 9.27 | 1.66 | 1.55E-02 |
| 218976_at | DNAJC12 | DnaJ (Hsp40) homolog, subfamily C, member 12 | unknown | other | 3.85 | 1.66 | 2.11E-03 |
| 232035_at | HIST1H4H | histone cluster 1, H4h | Nucleus | other | 4.71 | 1.67 | 1.72E-04 |
| 213519_s_at | LAMA2 | laminin, alpha 2 | Extracellular Space | other | 7.57 | 1.67 | 7.04E-03 |
| 208490_x_at | HIST1H2BF | histone cluster 1, H2bf | unknown | other | 6.91 | 1.67 | 1.19E-03 |
| 214708_at | SNTB1 | syntrophin, beta 1 (dystrophin-associated protein A1, 59kDa, basic component 1) | Plasma Membrane | other | 4.54 | 1.68 | 2.19E-03 |
| 228762_at | LFNG | LFNG O-fucosylpeptide 3-beta-N-acetylglucosaminyltransferase | Cytoplasm | enzyme | 6.11 | 1.68 | 2.18E-04 |
| 208978_at | CRIP2 | cysteine-rich protein 2 | Plasma Membrane | other | 8.58 | 1.69 | 3.31E-05 |
| 223279_s_at | UACA | uveal autoantigen with coiled-coil domains and ankyrin repeats | Cytoplasm | other | 8.46 | 1.69 | 2.55E-02 |
| 209937_at | TM4SF4 | transmembrane 4 L six family member 4 | Plasma Membrane | other | 4.73 | 1.69 | 1.27E-02 |
| 215011_at | SNHG3 | small nucleolar RNA host gene 3 (non-protein coding) | Nucleus | other | 4.97 | 1.69 | 2.01E-03 |
| 210090_at | ARC | activity-regulated cytoskeleton-associated protein | Cytoplasm | other | 4.43 | 1.69 | 2.30E-03 |
| 227270_at | FAM200B | family with sequence similarity 200, member B | unknown | other | 7.86 | 1.69 | 8.19E-05 |
| 204736_s_at | CSPG4 | chondroitin sulfate proteoglycan 4 | Plasma Membrane | other | 6.09 | 1.69 | 7.90E-03 |
| 203397_s_at | GALNT3 | UDP-N-acetyl-alpha-D-galactosamine:polypeptide N-acetylgalactosaminyltransferase 3 (GalNAc-T3) | Cytoplasm | enzyme | 3.65 | 1.70 | 8.78E-04 |
| 202825_at | SLC25A4 | solute carrier family 25 (mitochondrial carrier; adenine nucleotide translocator), member 4 | Cytoplasm | transporter | 8.18 | 1.71 | 2.67E-04 |
| 40560_at | TBX2 | T-box 2 | Nucleus | transcription regulator | 6.25 | 1.71 | 2.27E-02 |
| 211026_s_at | MGLL | monoglyceride lipase | Plasma Membrane | enzyme | 10.24 | 1.71 | 3.95E-02 |
| 204821_at | BTN3A3 | butyrophilin, subfamily 3, member A3 | unknown | other | 7.17 | 1.71 | 1.15E-03 |
| 212098_at | LOC151162 /// MGAT5 | hypothetical LOC151162 /// mannosyl (alpha-1,6-)-glycoprotein beta-1,6-N-acetyl-glucosaminyltransferase | Cytoplasm | enzyme | 8.85 | 1.72 | 1.32E-04 |
| 227900_at | CBLB | Cas-Br-M (murine) ecotropic retroviral transforming sequence b | Nucleus | other | 7.15 | 1.72 | 1.85E-02 |
| 208712_at | CCND1 | cyclin D1 | Nucleus | other | 10.85 | 1.72 | 1.59E-02 |
| 201341_at | ENC1 | ectodermal-neural cortex 1 (with BTB-like domain) | Nucleus | peptidase | 8.93 | 1.72 | 3.33E-02 |
| 211966_at | COL4A2 | collagen, type IV, alpha 2 | Extracellular Space | other | 7.36 | 1.72 | 2.20E-02 |
| 206224_at | CST1 | cystatin SN | unknown | other | 4.55 | 1.73 | 4.21E-02 |
| 205117_at | FGF1 | fibroblast growth factor 1 (acidic) | Extracellular Space | growth factor | 6.87 | 1.73 | 6.23E-04 |
| 213273_at | ODZ4 | odz, odd Oz/ten-m homolog 4 (Drosophila) | unknown | other | 5.09 | 1.73 | 7.53E-03 |
| 209911_x_at | HIST1H2BD | histone cluster 1, H2bd | Nucleus | other | 8.01 | 1.73 | 3.07E-03 |
| 209803_s_at | PHLDA2 | pleckstrin homology-like domain, family A, member 2 | Cytoplasm | other | 9.20 | 1.74 | 1.10E-02 |
| 204720_s_at | DNAJC6 | DnaJ (Hsp40) homolog, subfamily C, member 6 | Cytoplasm | other | 5.91 | 1.74 | 3.13E-02 |
| 229256_at | PGM2L1 | phosphoglucomutase 2-like 1 | unknown | enzyme | 6.38 | 1.74 | 7.76E-03 |
| 204491_at | PDE4D | phosphodiesterase 4D, cAMP-specific (phosphodiesterase E3 dunce homolog, Drosophila) | Cytoplasm | enzyme | 7.39 | 1.74 | 2.48E-03 |
| 210335_at | RASSF9 | Ras association (RalGDS/AF-6) domain family (N-terminal) member 9 | Cytoplasm | transporter | 4.67 | 1.74 | 1.85E-05 |
| 1555007_s_at | WDR66 | WD repeat domain 66 | unknown | other | 4.97 | 1.75 | 7.20E-04 |
| 221884_at | MECOM | MDS1 and EVI1 complex locus | Nucleus | transcription regulator | 5.05 | 1.75 | 1.26E-02 |
| 202747_s_at | ITM2A | integral membrane protein 2A | Plasma Membrane | other | 4.89 | 1.75 | 2.22E-02 |
| 202149_at | NEDD9 | neural precursor cell expressed, developmentally down-regulated 9 | Nucleus | other | 8.44 | 1.75 | 4.75E-02 |
| 1555960_at | HINT1 | histidine triad nucleotide binding protein 1 | Nucleus | enzyme | 5.88 | 1.75 | 2.30E-03 |
| 227828_s_at | FAM176A | family with sequence similarity 176, member A | unknown | other | 9.02 | 1.76 | 3.27E-02 |
| 232034_at | LOC203274 | hypothetical protein LOC203274 | unknown | other | 4.16 | 1.76 | 2.49E-03 |
| 214455_at | HIST1H2BC | histone cluster 1, H2bc | Nucleus | other | 4.48 | 1.77 | 4.47E-02 |
| 227196_at | RHPN2 | rhophilin, Rho GTPase binding protein 2 | Cytoplasm | other | 4.43 | 1.77 | 7.92E-04 |
| 227337_at | ANKRD37 | ankyrin repeat domain 37 | unknown | other | 7.43 | 1.77 | 6.95E-03 |
| 1555731_a_at | AP1S3 | adaptor-related protein complex 1, sigma 3 subunit | Cytoplasm | transporter | 3.80 | 1.78 | 7.14E-04 |
| 231380_at | C8orf34 | chromosome 8 open reading frame 34 | unknown | other | 5.12 | 1.78 | 5.38E-03 |
| 226069_at | PRICKLE1 | prickle homolog 1 (Drosophila) | Nucleus | other | 6.52 | 1.78 | 3.66E-03 |
| 232287_at | PGBD3 | piggyBac transposable element derived 3 | unknown | other | 6.963 | 1.79 | 9.84E-05 |
| 223658_at | KCNK6 | potassium channel, subfamily K, member 6 | Plasma Membrane | ion channel | 6.50 | 1.79 | 3.30E-04 |
| 219973_at | ARSJ | arylsulfatase family, member J | Extracellular Space | enzyme | 8.07 | 1.80 | 6.75E-03 |
| 239336_at | THBS1 | thrombospondin 1 | Extracellular Space | other | 8.03 | 1.80 | 3.42E-02 |
| 227371_at | BAIAP2L1 | BAI1-associated protein 2-like 1 | Cytoplasm | other | 4.94 | 1.80 | 4.66E-02 |
| 204471_at | GAP43 | growth associated protein 43 | Plasma Membrane | other | 3.67 | 1.80 | 1.39E-02 |
| 203502_at | BPGM | 2,3-bisphosphoglycerate mutase | unknown | phosphatase | 7.79 | 1.80 | 2.95E-04 |
| 207347_at | ERCC6 | excision repair cross-complementing rodent repair deficiency, complementation group 6 | Nucleus | transcription regulator | 5.17 | 1.81 | 8.27E-04 |
| 235518_at | SLC8A1 | solute carrier family 8 (sodium/calcium exchanger), member 1 | Plasma Membrane | transporter | 7.40 | 1.81 | 4.94E-02 |
| 201920_at | SLC20A1 | solute carrier family 20 (phosphate transporter), member 1 | Plasma Membrane | transporter | 10.27 | 1.81 | 2.19E-02 |
| 227705_at | TCEAL7 | transcription elongation factor A (SII)-like 7 | unknown | other | 8.52 | 1.81 | 9.37E-03 |
| 223349_s_at | BOK | BCL2-related ovarian killer | unknown | other | 7.27 | 1.82 | 1.13E-02 |
| 235337_at | SERTAD4 | SERTA domain containing 4 | unknown | other | 3.20 | 1.82 | 3.35E-05 |
| 230051_at | C10orf47 | chromosome 10 open reading frame 47 | unknown | other | 4.42 | 1.83 | 4.53E-03 |
| 206766_at | ITGA10 | integrin, alpha 10 | Plasma Membrane | other | 5.99 | 1.83 | 1.30E-02 |
| 223631_s_at | C19orf33 | chromosome 19 open reading frame 33 | Nucleus | other | 3.94 | 1.84 | 1.97E-02 |
| 1553565_s_at | DDAH1 | dimethylarginine dimethylaminohydrolase 1 | Cytoplasm | enzyme | 8.06 | 1.84 | 9.95E-03 |
| 222809_x_at | CCDC85C | coiled-coil domain containing 85C | unknown | other | 6.15 | 1.84 | 4.00E-02 |
| 212909_at | LYPD1 | LY6/PLAUR domain containing 1 | Plasma Membrane | G-protein coupled receptor | 4.17 | 1.84 | 7.28E-03 |
| 223427_s_at | EPB41L4B | erythrocyte membrane protein band 4.1 like 4B | unknown | transporter | 3.71 | 1.84 | 2.71E-02 |
| 205563_at | KISS1 | KiSS-1 metastasis-suppressor | Cytoplasm | other | 4.79 | 1.84 | 1.11E-02 |
| 229059_at | C9orf109 /// C9orf110 | chromosome 9 open reading frame 109 /// chromosome 9 open reading frame 110 | unknown | other | 4.39 | 1.84 | 2.11E-02 |
| 209468_at | LRP5 | low density lipoprotein receptor-related protein 5 | Plasma Membrane | other | 6.03 | 1.86 | 1.52E-03 |
| 212654_at | TPM2 | tropomyosin 2 (beta) | Cytoplasm | other | 8.29 | 1.86 | 3.19E-03 |
| 200884_at | CKB | creatine kinase, brain | Cytoplasm | kinase | 8.02 | 1.86 | 9.27E-03 |
| 210387_at | HIST1H2BG | histone cluster 1, H2bg | Nucleus | other | 4.28 | 1.86 | 7.89E-03 |
| 242396_at | LOC644192 | Hypothetical LOC644192 | unknown | other | 3.50 | 1.87 | 3.72E-03 |
| 208483_x_at | KRT33A | keratin 33A | Cytoplasm | other | 6.19 | 1.87 | 1.95E-03 |
| 209652_s_at | PGF | placental growth factor | Extracellular Space | growth factor | 8.31 | 1.87 | 3.83E-02 |
| 219773_at | NOX4 | NADPH oxidase 4 | Cytoplasm | enzyme | 4.44 | 1.87 | 2.47E-03 |
| 203499_at | EPHA2 | EPH receptor A2 | Plasma Membrane | kinase | 5.58 | 1.88 | 1.17E-02 |
| 212472_at | MICAL2 | microtubule associated monoxygenase, calponin and LIM domain containing 2 | Cytoplasm | other | 9.04 | 1.88 | 2.67E-02 |
| 208998_at | UCP2 | uncoupling protein 2 (mitochondrial, proton carrier) | Cytoplasm | transporter | 5.75 | 1.88 | 4.39E-02 |
| 205659_at | HDAC9 | histone deacetylase 9 | Nucleus | transcription regulator | 6.08 | 1.89 | 3.01E-02 |
| 1552309_a_at | NEXN | nexilin (F actin binding protein) | Plasma Membrane | other | 9.04 | 1.89 | 2.84E-04 |
| 205483_s_at | ISG15 | ISG15 ubiquitin-like modifier | Extracellular Space | other | 9.08 | 1.89 | 9.64E-05 |
| 212192_at | KCTD12 | potassium channel tetramerisation domain containing 12 | unknown | ion channel | 9.66 | 1.89 | 4.78E-02 |
| 216184_s_at | RIMS1 | regulating synaptic membrane exocytosis 1 | Cytoplasm | enzyme | 4.61 | 1.90 | 6.99E-04 |
| 219655_at | C7orf10 | chromosome 7 open reading frame 10 | unknown | other | 6.89 | 1.90 | 5.22E-06 |
| 228253_at | LOXL3 | lysyl oxidase-like 3 | Extracellular Space | enzyme | 8.47 | 1.90 | 1.84E-05 |
| 201058_s_at | MYL9 | myosin, light chain 9, regulatory | Cytoplasm | other | 11.58 | 1.90 | 4.91E-03 |
| 222771_s_at | MYEF2 | myelin expression factor 2 | Nucleus | transcription regulator | 4.01 | 1.91 | 4.88E-02 |
| 203153_at | IFIT1 | interferon-induced protein with tetratricopeptide repeats 1 | Cytoplasm | other | 7.90 | 1.91 | 1.27E-02 |
| 214767_s_at | HSPB6 | heat shock protein, alpha-crystallin-related, B6 | Cytoplasm | other | 7.02 | 1.91 | 4.88E-03 |
| 228875_at | FAM162B | family with sequence similarity 162, member B | unknown | other | 3.48 | 1.91 | 2.74E-02 |
| 206029_at | ANKRD1 | ankyrin repeat domain 1 (cardiac muscle) | Cytoplasm | transcription regulator | 3.32 | 1.92 | 1.75E-04 |
| 219474_at | C3orf52 | chromosome 3 open reading frame 52 | unknown | other | 5.81 | 1.92 | 4.26E-02 |
| 209211_at | KLF5 | Kruppel-like factor 5 (intestinal) | Nucleus | transcription regulator | 3.69 | 1.92 | 1.96E-02 |
| 225168_at | FRMD4A | FERM domain containing 4A | unknown | other | 7.59 | 1.93 | 2.84E-03 |
| 210605_s_at | MFGE8 | milk fat globule-EGF factor 8 protein | Extracellular Space | other | 8.32 | 1.93 | 2.57E-02 |
| 227606_s_at | STAMBPL1 | STAM binding protein-like 1 | unknown | other | 6.82 | 1.93 | 4.48E-02 |
| 224325_at | FZD8 | frizzled homolog 8 (Drosophila) | Plasma Membrane | G-protein coupled receptor | 6.45 | 1.94 | 2.28E-02 |
| 226757_at | IFIT2 | interferon-induced protein with tetratricopeptide repeats 2 | unknown | other | 7.00 | 1.94 | 6.24E-03 |
| 207034_s_at | GLI2 | GLI family zinc finger 2 | Nucleus | transcription regulator | 6.33 | 1.94 | 2.17E-02 |
| 220765_s_at | LIMS2 | LIM and senescent cell antigen-like domains 2 | Cytoplasm | other | 7.02 | 1.94 | 1.73E-03 |
| 210654_at | TNFRSF10D | tumor necrosis factor receptor superfamily, member 10d, decoy with truncated death domain | Plasma Membrane | transmembrane receptor | 6.83 | 1.94 | 1.85E-03 |
| 223092_at | ANKH | ankylosis, progressive homolog (mouse) | Plasma Membrane | transporter | 9.27 | 1.94 | 5.60E-03 |
| 219282_s_at | TRPV2 | transient receptor potential cation channel, subfamily V, member 2 | Plasma Membrane | ion channel | 8.69 | 1.95 | 5.07E-03 |
| 230720_at | RNF182 | ring finger protein 182 | unknown | other | 6.57 | 1.95 | 4.52E-03 |
| 202923_s_at | GCLC | glutamate-cysteine ligase, catalytic subunit | Cytoplasm | enzyme | 7.55 | 1.96 | 1.05E-02 |
| 219522_at | FJX1 | four jointed box 1 (Drosophila) | Extracellular Space | other | 8.33 | 1.97 | 3.87E-03 |
| 203217_s_at | 203217_s_at | ST3 beta-galactoside alpha-2,3-sialyltransferase 5 | Cytoplasm | enzyme | 7.34 | 1.98 | 2.33E-05 |
| 1552658_a_at | NAV3 | neuron navigator 3 | unknown | other | 7.93 | 1.98 | 1.38E-03 |
| 229778_at | C12orf39 | chromosome 12 open reading frame 39 | Extracellular Space | other | 3.19 | 1.99 | 7.79E-03 |
| 211071_s_at | MLLT11 | myeloid/lymphoid or mixed-lineage leukemia (trithorax homolog, Drosophila); translocated to, 11 | unknown | other | 9.30 | 1.99 | 5.63E-03 |
| 201387_s_at | UCHL1 | ubiquitin carboxyl-terminal esterase L1 (ubiquitin thiolesterase) | Cytoplasm | peptidase | 11.03 | 1.99 | 1.01E-02 |
| 226899_at | UNC5B | unc-5 homolog B (C. elegans) | Plasma Membrane | transmembrane receptor | 6.43 | 2.01 | 2.26E-02 |
| 228360_at | LYPD6B | LY6/PLAUR domain containing 6B | unknown | other | 7.70 | 2.01 | 3.13E-03 |
| 206953_s_at | LPHN2 | latrophilin 2 | Plasma Membrane | G-protein coupled receptor | 8.34 | 2.02 | 3.77E-02 |
| 201288_at | ARHGDIB | Rho GDP dissociation inhibitor (GDI) beta | Cytoplasm | other | 6.77 | 2.02 | 1.36E-02 |
| 217841_s_at | PPME1 | protein phosphatase methylesterase 1 | unknown | enzyme | 8.83 | 2.03 | 1.95E-04 |
| 203951_at | CNN1 | calponin 1, basic, smooth muscle | Cytoplasm | other | 8.49 | 2.04 | 4.80E-03 |
| 219956_at | GALNT6 | UDP-N-acetyl-alpha-D-galactosamine:polypeptide N-acetylgalactosaminyltransferase 6 (GalNAc-T6) | Cytoplasm | enzyme | 6.40 | 2.04 | 1.22E-02 |
| 200923_at | LGALS3BP | lectin, galactoside-binding, soluble, 3 binding protein | Plasma Membrane | transmembrane receptor | 8.72 | 2.04 | 4.86E-04 |
| 229461_x_at | NEGR1 | neuronal growth regulator 1 | Extracellular Space | other | 7.86 | 2.04 | 5.49E-04 |
| 203242_s_at | PDLIM5 | PDZ and LIM domain 5 | Cytoplasm | other | 8.31 | 2.05 | 3.25E-05 |
| 220976_s_at | KRTAP1-1 | keratin associated protein 1-1 | unknown | other | 7.97 | 2.06 | 4.39E-02 |
| 203810_at | DNAJB4 | DnaJ (Hsp40) homolog, subfamily B, member 4 | Nucleus | other | 9.13 | 2.06 | 7.55E-03 |
| 214761_at | ZNF423 | zinc finger protein 423 | Nucleus | transcription regulator | 6.01 | 2.06 | 7.68E-03 |
| 213664_at | SLC1A1 | solute carrier family 1 (neuronal/epithelial high affinity glutamate transporter, system Xag), member 1 | Plasma Membrane | transporter | 8.99 | 2.07 | 1.37E-03 |
| 219179_at | DACT1 | dapper, antagonist of beta-catenin, homolog 1 (Xenopus laevis) | Cytoplasm | other | 8.41 | 2.07 | 1.31E-02 |
| 217234_s_at | EZR | ezrin | Plasma Membrane | other | 7.16 | 2.09 | 4.34E-02 |
| 227198_at | AFF3 | AF4/FMR2 family, member 3 | Nucleus | transcription regulator | 4.86 | 2.11 | 1.92E-02 |
| 201615_x_at | CALD1 | caldesmon 1 | Cytoplasm | other | 10.30 | 2.11 | 1.06E-03 |
| 209948_at | KCNMB1 | potassium large conductance calcium-activated channel, subfamily M, beta member 1 | Plasma Membrane | ion channel | 4.64 | 2.12 | 2.91E-02 |
| 228949_at | WLS | wntless homolog (Drosophila) | unknown | other | 7.47 | 2.12 | 1.63E-03 |
| 226278_at | SVIP | small VCP/p97-interacting protein | Cytoplasm | other | 6.71 | 2.13 | 2.07E-03 |
| 202719_s_at | TES | testis derived transcript (3 LIM domains) | Plasma Membrane | other | 7.93 | 2.13 | 1.20E-03 |
| 201010_s_at | TXNIP | thioredoxin interacting protein | Cytoplasm | other | 8.57 | 2.13 | 1.02E-02 |
| 227875_at | KLHL13 | kelch-like 13 (Drosophila) | unknown | other | 4.25 | 2.14 | 1.51E-02 |
| 209789_at | CORO2B | coronin, actin binding protein, 2B | unknown | other | 6.01 | 2.14 | 1.26E-02 |
| 228224_at | PRELP | proline/arginine-rich end leucine-rich repeat protein | Extracellular Space | other | 7.00 | 2.14 | 6.52E-04 |
| 214467_at | GPR65 | G protein-coupled receptor 65 | Plasma Membrane | G-protein coupled receptor | 3.62 | 2.14 | 1.90E-02 |
| 205825_at | PCSK1 | proprotein convertase subtilisin/kexin type 1 | Extracellular Space | peptidase | 3.88 | 2.15 | 3.15E-02 |
| 1555724_s_at | TAGLN | transgelin | Cytoplasm | other | 12.69 | 2.15 | 2.27E-03 |
| 201195_s_at | SLC7A5 | solute carrier family 7 (cationic amino acid transporter, y+ system), member 5 | Plasma Membrane | transporter | 7.95 | 2.15 | 1.72E-02 |
| 212314_at | SEL1L3 | sel-1 suppressor of lin-12-like 3 (C. elegans) | unknown | other | 8.30 | 2.16 | 7.55E-04 |
| 228245_s_at | OVOS /// OVOS2 | ovostatin /// ovostatin 2 | unknown | other | 4.03 | 2.17 | 3.53E-02 |
| 205266_at | LIF | leukemia inhibitory factor (cholinergic differentiation factor) | Extracellular Space | cytokine | 6.14 | 2.18 | 4.54E-03 |
| 213075_at | OLFML2A | olfactomedin-like 2A | unknown | other | 5.44 | 2.19 | 1.91E-03 |
| 219829_at | ITGB1BP2 | integrin beta 1 binding protein (melusin) 2 | unknown | other | 5.24 | 2.20 | 3.71E-05 |
| 209723_at | SERPINB9 | serpin peptidase inhibitor, clade B (ovalbumin), member 9 | Cytoplasm | other | 6.27 | 2.22 | 1.40E-04 |
| 214091_s_at | GPX3 | glutathione peroxidase 3 (plasma) | Extracellular Space | enzyme | 6.49 | 2.22 | 2.07E-02 |
| 238029_s_at | SLC16A14 | solute carrier family 16, member 14 (monocarboxylic acid transporter 14) | unknown | other | 3.35 | 2.23 | 6.09E-03 |
| 225525_at | KIAA1671 | KIAA1671 | unknown | other | 5.62 | 2.25 | 2.47E-03 |
| 235228_at | CCDC85A | coiled-coil domain containing 85A | unknown | other | 7.41 | 2.25 | 4.22E-02 |
| 210715_s_at | SPINT2 | serine peptidase inhibitor, Kunitz type, 2 | Extracellular Space | other | 5.11 | 2.26 | 1.45E-05 |
| 201250_s_at | SLC2A1 | solute carrier family 2 (facilitated glucose transporter), member 1 | Plasma Membrane | transporter | 7.20 | 2.28 | 2.06E-04 |
| 221577_x_at | GDF15 /// LOC100292463 | growth differentiation factor 15 /// similar to growth differentiation factor 15 | Extracellular Space | growth factor | 6.74 | 2.28 | 4.22E-02 |
| 209016_s_at | KRT7 | keratin 7 | Cytoplasm | other | 9.10 | 2.29 | 1.77E-02 |
| 1559277_at | FLJ35700 | hypothetical protein FLJ35700 | unknown | other | 3.68 | 2.31 | 6.71E-06 |
| 233533_at | KRTAP1-5 | keratin associated protein 1-5 | unknown | other | 10.52 | 2.32 | 3.84E-02 |
| 227948_at | FGD4 | FYVE, RhoGEF and PH domain containing 4 | Cytoplasm | other | 6.29 | 2.33 | 2.41E-02 |
| 213421_x_at | PRSS3 | protease, serine, 3 | Extracellular Space | peptidase | 5.53 | 2.33 | 3.04E-02 |
| 227566_at | NTM | neurotrimin | Plasma Membrane | other | 8.56 | 2.35 | 1.53E-03 |
| 209763_at | CHRDL1 | chordin-like 1 | Extracellular Space | other | 5.38 | 2.37 | 4.00E-02 |
| 218736_s_at | PALMD | palmdelphin | unknown | other | 4.78 | 2.40 | 1.21E-02 |
| 205893_at | NLGN1 | neuroligin 1 | Plasma Membrane | enzyme | 5.30 | 2.41 | 1.95E-03 |
| 205066_s_at | ENPP1 | ectonucleotide pyrophosphatase/phosphodiesterase 1 | Plasma Membrane | enzyme | 7.32 | 2.41 | 2.41E-02 |
| 228737_at | TOX2 | TOX high mobility group box family member 2 | Nucleus | transcription regulator | 6.31 | 2.42 | 2.91E-02 |
| 205715_at | BST1 | bone marrow stromal cell antigen 1 | Plasma Membrane | enzyme | 8.28 | 2.45 | 1.92E-04 |
| 231807_at | KIAA1217 | KIAA1217 | Cytoplasm | other | 7.03 | 2.46 | 8.66E-03 |
| 206622_at | TRH | thyrotropin-releasing hormone | Extracellular Space | other | 5.35 | 2.48 | 6.79E-03 |
| 205590_at | RASGRP1 | RAS guanyl releasing protein 1 (calcium and DAG-regulated) | Cytoplasm | other | 4.07 | 2.48 | 2.09E-02 |
| 219506_at | C1orf54 | chromosome 1 open reading frame 54 | unknown | other | 8.55 | 2.48 | 1.28E-04 |
| 205532_s_at | CDH6 | cadherin 6, type 2, K-cadherin (fetal kidney) | Plasma Membrane | other | 4.28 | 2.49 | 7.00E-03 |
| 229893_at | FRMD3 | FERM domain containing 3 | unknown | other | 6.55 | 2.49 | 4.49E-02 |
| 219263_at | RNF128 | ring finger protein 128 | Cytoplasm | enzyme | 4.05 | 2.50 | 5.75E-03 |
| 200621_at | CSRP1 | cysteine and glycine-rich protein 1 | Nucleus | other | 10.86 | 2.51 | 2.00E-04 |
| 211980_at | COL4A1 | collagen, type IV, alpha 1 | Extracellular Space | other | 9.14 | 2.51 | 7.73E-03 |
| 216268_s_at | JAG1 | jagged 1 (Alagille syndrome) | Extracellular Space | growth factor | 7.45 | 2.51 | 6.80E-03 |
| 228121_at | TGFB2 | transforming growth factor, beta 2 | Extracellular Space | growth factor | 5.02 | 2.52 | 1.56E-02 |
| 204035_at | SCG2 | secretogranin II | Extracellular Space | cytokine | 4.34 | 2.53 | 1.90E-03 |
| 209047_at | AQP1 | aquaporin 1 (Colton blood group) | Plasma Membrane | transporter | 6.09 | 2.55 | 4.81E-03 |
| 204914_s_at | SOX11 | SRY (sex determining region Y)-box 11 | Nucleus | transcription regulator | 5.26 | 2.55 | 2.25E-03 |
| 226415_at | VAT1L | vesicle amine transport protein 1 homolog (T. californica)-like | unknown | enzyme | 6.22 | 2.55 | 2.70E-04 |
| 213620_s_at | ICAM2 | intercellular adhesion molecule 2 | Plasma Membrane | other | 6.20 | 2.57 | 2.99E-02 |
| 213222_at | PLCB1 | phospholipase C, beta 1 (phosphoinositide-specific) | Cytoplasm | enzyme | 7.04 | 2.60 | 2.68E-03 |
| 206969_at | KRT34 | keratin 34 | Cytoplasm | other | 8.31 | 2.62 | 4.94E-03 |
| 215870_s_at | PLA2G5 | phospholipase A2, group V | Extracellular Space | enzyme | 4.21 | 2.62 | 1.12E-04 |
| 218974_at | SOBP | sine oculis binding protein homolog (Drosophila) | unknown | other | 6.21 | 2.65 | 5.93E-05 |
| 227889_at | LPCAT2 | lysophosphatidylcholine acyltransferase 2 | Cytoplasm | enzyme | 6.52 | 2.67 | 2.80E-03 |
| 209875_s_at | SPP1 | secreted phosphoprotein 1 | Extracellular Space | cytokine | 6.32 | 2.68 | 2.60E-03 |
| 214043_at | PTPRD | protein tyrosine phosphatase, receptor type, D | Plasma Membrane | phosphatase | 3.66 | 2.68 | 3.95E-03 |
| 230109_at | PDE7B | phosphodiesterase 7B | Cytoplasm | enzyme | 7.46 | 2.70 | 8.14E-04 |
| 224396_s_at | ASPN | asporin | Extracellular Space | other | 4.37 | 2.73 | 1.45E-02 |
| 206128_at | ADRA2C | adrenergic, alpha-2C-, receptor | Plasma Membrane | G-protein coupled receptor | 6.48 | 2.73 | 6.34E-04 |
| 224435_at | C10orf58 | chromosome 10 open reading frame 58 | Extracellular Space | other | 5.51 | 2.74 | 6.98E-03 |
| 226911_at | EGFLAM | EGF-like, fibronectin type III and laminin G domains | unknown | other | 5.62 | 2.74 | 9.15E-03 |
| 210511_s_at | INHBA | inhibin, beta A | Extracellular Space | growth factor | 9.66 | 2.75 | 1.10E-04 |
| 219140_s_at | RBP4 | retinol binding protein 4, plasma | Extracellular Space | transporter | 4.63 | 2.76 | 1.11E-02 |
| 205080_at | RARB | retinoic acid receptor, beta | Nucleus | ligand-dependent nuclear receptor | 6.23 | 2.76 | 4.36E-02 |
| 206315_at | CRLF1 | cytokine receptor-like factor 1 | Extracellular Space | other | 7.96 | 2.79 | 4.59E-03 |
| 222717_at | SDPR | serum deprivation response | Plasma Membrane | other | 5.58 | 2.80 | 6.07E-03 |
| 220108_at | GNA14 | guanine nucleotide binding protein (G protein), alpha 14 | Plasma Membrane | enzyme | 4.88 | 2.80 | 4.38E-02 |
| 220389_at | CCDC81 | coiled-coil domain containing 81 | unknown | other | 4.36 | 2.81 | 5.91E-04 |
| 227282_at | PCDH19 | protocadherin 19 | unknown | other | 4.56 | 2.81 | 1.53E-06 |
| 213906_at | MYBL1 | v-myb myeloblastosis viral oncogene homolog (avian)-like 1 | Nucleus | transcription regulator | 7.47 | 2.82 | 4.79E-02 |
| 205990_s_at | WNT5A | wingless-type MMTV integration site family, member 5A | Extracellular Space | other | 9.42 | 2.82 | 8.41E-04 |
| 219049_at | CSGALNACT1 | chondroitin sulfate N-acetylgalactosaminyltransferase 1 | Cytoplasm | enzyme | 6.53 | 2.82 | 4.00E-03 |
| 206343_s_at | NRG1 | neuregulin 1 | Extracellular Space | growth factor | 7.83 | 2.85 | 1.28E-02 |
| 203786_s_at | TPD52L1 | tumor protein D52-like 1 | Cytoplasm | other | 7.62 | 2.85 | 7.22E-03 |
| 203766_s_at | LMOD1 | leiomodin 1 (smooth muscle) | Cytoplasm | other | 7.59 | 2.85 | 3.30E-03 |
| 228618_at | PEAR1 | platelet endothelial aggregation receptor 1 | unknown | other | 6.84 | 2.86 | 1.23E-03 |
| 200606_at | DSP | desmoplakin | Plasma Membrane | other | 9.20 | 2.89 | 1.76E-03 |
| 200974_at | ACTA2 | actin, alpha 2, smooth muscle, aorta | Cytoplasm | other | 11.87 | 2.94 | 1.23E-03 |
| 227099_s_at | AG2 | protein Ag2 homolog | unknown | other | 8.10 | 2.96 | 2.90E-02 |
| 206290_s_at | RGS7 | regulator of G-protein signaling 7 | Cytoplasm | enzyme | 5.36 | 2.96 | 3.32E-03 |
| 205923_at | RELN | reelin | Extracellular Space | peptidase | 6.21 | 2.97 | 9.42E-03 |
| 227410_at | FAM43A | family with sequence similarity 43, member A | unknown | other | 7.99 | 2.97 | 6.39E-03 |
| 204273_at | EDNRB | endothelin receptor type B | Plasma Membrane | G-protein coupled receptor | 3.84 | 3.00 | 1.05E-02 |
| 208025_s_at | HMGA2 | high mobility group AT-hook 2 | Nucleus | other | 7.37 | 3.04 | 1.71E-02 |
| 235489_at | RHOJ | ras homolog gene family, member J | Cytoplasm | enzyme | 4.23 | 3.08 | 1.43E-04 |
| 205016_at | TGFA | transforming growth factor, alpha | Extracellular Space | growth factor | 4.99 | 3.11 | 4.02E-02 |
| 203108_at | GPRC5A | G protein-coupled receptor, family C, group 5, member A | Plasma Membrane | G-protein coupled receptor | 6.82 | 3.18 | 8.56E-04 |
| 203989_x_at | F2R | coagulation factor II (thrombin) receptor | Plasma Membrane | G-protein coupled receptor | 8.11 | 3.20 | 2.43E-04 |
| 209841_s_at | LRRN3 | leucine rich repeat neuronal 3 | Extracellular Space | other | 5.75 | 3.21 | 1.65E-04 |
| 205258_at | INHBB | inhibin, beta B | Extracellular Space | growth factor | 5.88 | 3.28 | 2.79E-07 |
| 209170_s_at | GPM6B | glycoprotein M6B | Plasma Membrane | other | 6.00 | 3.29 | 2.67E-04 |
| 204931_at | TCF21 | transcription factor 21 | Nucleus | transcription regulator | 5.27 | 3.29 | 6.47E-05 |
| 205700_at | HSD17B6 | hydroxysteroid (17-beta) dehydrogenase 6 homolog (mouse) | unknown | enzyme | 4.46 | 3.31 | 3.18E-04 |
| 206382_s_at | BDNF | brain-derived neurotrophic factor | Extracellular Space | growth factor | 8.02 | 3.31 | 6.35E-03 |
| 1556029_s_at | NMNAT2 | nicotinamide nucleotide adenylyltransferase 2 | Cytoplasm | enzyme | 4.19 | 3.39 | 1.88E-04 |
| 227243_s_at | EBF3 | early B-cell factor 3 | Nucleus | other | 7.02 | 3.40 | 3.27E-04 |
| 230214_at | MRVI1 | murine retrovirus integration site 1 homolog | Cytoplasm | other | 6.97 | 3.42 | 2.75E-02 |
| 227236_at | TSPAN2 | tetraspanin 2 | unknown | other | 5.25 | 3.46 | 1.25E-03 |
| 214974_x_at | CXCL5 | chemokine (C-X-C motif) ligand 5 | Extracellular Space | cytokine | 6.53 | 3.59 | 2.97E-02 |
| 230493_at | SHISA2 | shisa homolog 2 (Xenopus laevis) | unknown | other | 4.76 | 3.60 | 1.44E-02 |
| 219073_s_at | OSBPL10 | oxysterol binding protein-like 10 | unknown | other | 4.69 | 3.63 | 7.85E-07 |
| 209395_at | CHI3L1 | chitinase 3-like 1 (cartilage glycoprotein-39) | Extracellular Space | enzyme | 4.70 | 3.64 | 1.77E-03 |
| 217757_at | A2M | alpha-2-macroglobulin | Extracellular Space | transporter | 6.98 | 3.67 | 2.88E-02 |
| 206488_s_at | CD36 | CD36 molecule (thrombospondin receptor) | Plasma Membrane | transmembrane receptor | 4.62 | 3.79 | 1.16E-02 |
| 213880_at | LGR5 | leucine-rich repeat-containing G protein-coupled receptor 5 | Plasma Membrane | G-protein coupled receptor | 3.90 | 3.91 | 6.93E-06 |
| 201162_at | IGFBP7 | insulin-like growth factor binding protein 7 | Extracellular Space | transporter | 11.50 | 3.93 | 3.87E-04 |
| 226244_at | CLEC14A | C-type lectin domain family 14, member A | unknown | other | 6.44 | 3.97 | 2.37E-04 |
| 203238_s_at | NOTCH3 | Notch homolog 3 (Drosophila) | Plasma Membrane | transcription regulator | 7.44 | 4.06 | 4.67E-03 |
| 207316_at | HAS1 | hyaluronan synthase 1 | Plasma Membrane | enzyme | 5.63 | 4.10 | 3.60E-05 |
| 205352_at | SERPINI1 | serpin peptidase inhibitor, clade I (neuroserpin), member 1 | Extracellular Space | other | 5.92 | 4.11 | 6.80E-06 |
| 209277_at | TFPI2 | tissue factor pathway inhibitor 2 | Extracellular Space | other | 7.36 | 4.16 | 8.47E-03 |
| 213496_at | LPPR4 | lipid phosphate phosphatase-related protein type 4 | Plasma Membrane | phosphatase | 5.99 | 4.16 | 2.33E-02 |
| 203881_s_at | DMD | dystrophin | Plasma Membrane | other | 4.54 | 4.19 | 1.12E-06 |
| 214111_at | OPCML | opioid binding protein/cell adhesion molecule-like | Plasma Membrane | transmembrane receptor | 5.93 | 4.24 | 1.31E-02 |
| 204465_s_at | INA | internexin neuronal intermediate filament protein, alpha | Cytoplasm | other | 6.62 | 4.28 | 5.76E-06 |
| 203980_at | FABP4 | fatty acid binding protein 4, adipocyte | Cytoplasm | transporter | 3.94 | 4.40 | 4.82E-03 |
| 211959_at | IGFBP5 | insulin-like growth factor binding protein 5 | Extracellular Space | other | 8.36 | 4.42 | 2.50E-02 |
| 204051_s_at | SFRP4 | secreted frizzled-related protein 4 | Plasma Membrane | transmembrane receptor | 5.32 | 4.58 | 6.24E-05 |
| 223253_at | EPDR1 | ependymin related protein 1 (zebrafish) | Nucleus | other | 8.45 | 4.63 | 5.92E-04 |
| 206163_at | MAB21L1 | mab-21-like 1 (C. elegans) | unknown | other | 5.62 | 4.71 | 4.70E-03 |
| 37892_at | COL11A1 | collagen, type XI, alpha 1 | Extracellular Space | other | 7.69 | 4.72 | 8.64E-03 |
| 206176_at | BMP6 | bone morphogenetic protein 6 | Extracellular Space | growth factor | 6.03 | 4.85 | 2.18E-03 |
| 223315_at | NTN4 | netrin 4 | Extracellular Space | other | 6.92 | 4.87 | 7.30E-04 |
| 201042_at | TGM2 | transglutaminase 2 (C polypeptide, protein-glutamine-gamma-glutamyltransferase) | Cytoplasm | enzyme | 7.91 | 4.91 | 3.58E-03 |
| 1562736_at | LHX9 | LIM homeobox 9 | Nucleus | transcription regulator | 3.95 | 5.10 | 6.09E-03 |
| 225728_at | SORBS2 | sorbin and SH3 domain containing 2 | Nucleus | other | 4.67 | 5.11 | 9.73E-05 |
| 228885_at | MAMDC2 | MAM domain containing 2 | Extracellular Space | other | 5.45 | 5.19 | 9.24E-04 |
| 221805_at | NEFL | neurofilament, light polypeptide | Cytoplasm | other | 5.99 | 5.33 | 3.30E-02 |
| 206201_s_at | MEOX2 | mesenchyme homeobox 2 | Nucleus | transcription regulator | 4.50 | 5.36 | 5.97E-04 |
| 225275_at | EDIL3 | EGF-like repeats and discoidin I-like domains 3 | Extracellular Space | other | 10.02 | 5.45 | 2.55E-03 |
| 219478_at | WFDC1 | WAP four-disulfide core domain 1 | Extracellular Space | other | 7.06 | 5.53 | 8.50E-03 |
| 239058_at | FOXC2 | Forkhead box C2 (MFH-1, mesenchyme forkhead 1) | Nucleus | transcription regulator | 7.69 | 5.61 | 2.92E-03 |
| 237206_at | MYOCD | myocardin | Nucleus | transcription regulator | 5.29 | 5.72 | 1.07E-05 |
| 202016_at | MEST | mesoderm specific transcript homolog (mouse) | Extracellular Space | peptidase | 6.86 | 5.80 | 1.86E-03 |
| 201858_s_at | SRGN | serglycin | Extracellular Space | other | 9.58 | 5.95 | 7.33E-05 |
| 205113_at | NEFM | neurofilament, medium polypeptide | Cytoplasm | other | 8.76 | 6.20 | 4.91E-03 |
| 230147_at | F2RL2 | coagulation factor II (thrombin) receptor-like 2 | Plasma Membrane | G-protein coupled receptor | 6.86 | 6.43 | 7.46E-05 |
| 201596_x_at | KRT18 | keratin 18 | Cytoplasm | other | 6.69 | 6.68 | 2.38E-05 |
| 201650_at | KRT19 | keratin 19 | Cytoplasm | other | 8.63 | 6.69 | 1.67E-02 |
| 209343_at | EFHD1 | EF-hand domain family, member D1 | unknown | other | 5.90 | 6.77 | 4.11E-06 |
| 205475_at | SCRG1 | stimulator of chondrogenesis 1 | Extracellular Space | other | 5.51 | 6.88 | 7.14E-05 |
| 228407_at | SCUBE3 | signal peptide, CUB domain, EGF-like 3 | Plasma Membrane | other | 9.31 | 6.92 | 3.64E-03 |
| 237466_s_at | HHIP | hedgehog interacting protein | Plasma Membrane | other | 4.21 | 7.00 | 6.21E-03 |
| 206204_at | GRB14 | growth factor receptor-bound protein 14 | Plasma Membrane | other | 4.71 | 7.27 | 1.38E-02 |
| 224646_x_at | H19 | H19, imprinted maternally expressed transcript (non-protein coding) | unknown | other | 6.01 | 7.37 | 2.22E-02 |
| 213791_at | PENK | proenkephalin | Extracellular Space | other | 7.98 | 8.49 | 7.53E-04 |
| 230895_at | HAPLN1 | hyaluronan and proteoglycan link protein 1 | Extracellular Space | other | 6.74 | 9.20 | 4.23E-03 |
| 215034_s_at | TM4SF1 | transmembrane 4 L six family member 1 | Plasma Membrane | other | 6.79 | 9.70 | 1.53E-02 |
| 227006_at | PPP1R14A | protein phosphatase 1, regulatory (inhibitor) subunit 14A | Cytoplasm | other | 6.52 | 10.68 | 1.52E-04 |

Gene expression differences in primary cultured skin fibroblasts from affected *LMNA^K542/K542N^* carriers compared to healthy *LMNA^K542/+^* heterozygotes and controls (with fold change >1.5 and p value <0.05). Signal intensity (A), fold-change (FC) and p-values are given for each gene along with Affymetrix probe set ID, gene symbol, gene name, cellular localization and type of the encoded protein.
