# Supplementary material for: Discordant Gene Expression Signatures and Related Phenotypic Differences in Lamin A- and A/C-Related Hutchinson-Gilford Progeria Syndrome (HGPS)
Source: PLoS One. 2011 Jun 27;6(6):e21433. doi: 10.1371/journal.pone.0021433 (PMC3124505; doi:10.1371/journal.pone.0021433)
Supplement: Table S2 — Validation of the microarray results with TaqMan Gene Expression Assays. (DOC) [file pone.0021433.s004.doc]

**Table S2. Validation of the microarray results with TaqMan Gene Expression Assays.**

| **Gene symbol** | **TaqMan Assay ID** | **Fold changes** | **p value** |
| --- | --- | --- | --- |
| FGF13 | Hs00182807_m1 | -4.53 | 3.2E-03 |
| TWIST2 | Hs02379973_s1 | -3.16 | 1.6E-02 |
| TWIST2 | Hs00382379_m1 | -2.86 | 1.6E-02 |
| PBX1 | Hs00231228_m1 | -2.64 | 4.5E-03 |
| AKR1C1 | Hs00413886_m1 | -2.45 | 3.5E-02 |
| TNFRSF11B | Hs00900360_m1 | -2.41 | 3.7E-02 |
| TNFRSF11B | Hs00900358_m1 | -2.27 | 5.2E-02 |
| CXCL12 | Hs00930455_m1 | -2.34 | 1.7E-02 |
| CXCL12 | Hs00171022_m1 | -2.26 | 4.1E-03 |
| NES | Hs00707120_s1 | 1.99 | 2.6E-02 |
| TAGLN | Hs00162558_m1 | 2.23 | 3.4E-02 |
| ENPP1 | Hs01054040_m1 | 3 | 6.5E-02 |
| ENPP1 | Hs01054049_m1 | 3.32 | 5.5E-02 |
| INHBA | Hs00170103_m1 | 3.42 | 1.2E-03 |
| WNT5A | Hs00998537_m1 | 3.98 | 2.6E-02 |
| IL1B | Hs01555410_m1 | 4.38 | 4.4E-02 |
| IL1B | Hs01555413_m1 | 4.5 | 4.6E-02 |
| IGFBP7 | Hs00266026_m1 | 4.8 | 2.0E-02 |
| EDIL3 | Hs00174781_m1 | 5.21 | 1.7E-02 |
| DMD | Hs01049460_m1 | 5.92 | 1.1E-02 |
| INHBB | Hs00173582_m1 | 6.42 | 1.8E-02 |
| SRGN | Hs01004159_m1 | 7.47 | 8.9E-03 |
| KRT18 | Hs01653110_s1 | 7.59 | 2.0E-02 |
| SPP1 | Hs00959010_m1 | 14.17 | 6.1E-03 |
