# Supplementary material for: Discordant Gene Expression Signatures and Related Phenotypic Differences in Lamin A- and A/C-Related Hutchinson-Gilford Progeria Syndrome (HGPS)
Source: PLoS One. 2011 Jun 27;6(6):e21433. doi: 10.1371/journal.pone.0021433 (PMC3124505; doi:10.1371/journal.pone.0021433)
Supplement: Table S3 — Common gene expression signatures of the LMNA K542N and G608G mutation. (A) Genes with concordant (matching) transcriptional patterns. (B) Genes with discordant (opposite) transcriptional patterns. (DOC) [file pone.0021433.s005.doc]

**Table S3. Common gene expression signatures of the *LMNA* K542N and G608G mutation.**

**A)** **Genes with concordant (matching) transcriptional patterns**

| **Gene symbol** | **Fold change** | | |
| --- | --- | --- | --- |
|  | ***LMNA^K542/K542N^*** | ***LMNA^G608G/+^* [28]** | ***LMNA^G608G/+^* [29]** |
| FGF13 | -6.46 | -3.60 |  |
| PLXNC1 | -5.67 |  | -11.68 |
| TWIST2 | -3.46 | -3.00 |  |
| DPT | -3.36 | -5.00 | -31.06 |
| DKFZP586K1520 | -3.28 | -2.20 |  |
| CCRL1 | -3.04 | -3.40 |  |
| MXRA5 (DKFZp564I1922) | -2.76 | -3.40 | -24.84 |
| RNF144A (RNF144) | -2.73 | -5.80 |  |
| BCL11B | -2.64 | -5.50 |  |
| BOC | -2.56 | -2.60 |  |
| DPP4 | -2.52 |  | -29.56 |
| COLEC12 | -2.44 | -2.70 |  |
| MME | -2.26 |  | -25.17 |
| PBX1 | -2.23 | -6.60 |  |
| IRX1 | -2.19 | -5.70 |  |
| SEPP1 | -2.13 |  | -25.74 |
| MAFB | -2.04 |  | -5.52 |
| KIAA1644 | -1.93 | -3.10 |  |
| ENPP2 | -1.89 |  | -33.63 |
| KCNS3 | -1.88 | -3.30 |  |
| STEAP1 (STEAP) | -1.83 | -4.30 |  |
| KBTBD11 (KIAA0711) | -1.72 | -3.60 |  |
| SLC38A5 | -1.69 | -2.00 |  |
| FOXF2 | -1.66 | -3.60 |  |
| SDC1 | -1.62 | -4.00 |  |
| SPOCK1 (SPOCK) | 1.50 | 2.10 |  |
| SLC7A11 | 1.54 | 6.00 |  |
| CCDC110 (MGC33607) | 1.58 | 3.10 |  |
| ENG | 1.64 | 2.80 |  |
| JUP | 1.65 | 3.50 |  |
| SNTB1 | 1.68 | 8.40 |  |
| GALNT3 | 1.70 | 13.50 |  |
| BTN3A3 | 1.71 | 2.10 |  |
| SLC25A4 | 1.71 | 2.90 |  |
| COL4A2 | 1.72 | 2.50 | 5.02 |
| PGBD3 | 1.79 | 2.60 |  |
| HDAC9 | 1.89 | 2.50 |  |
| C7orf10 | 1.90 | 3.60 |  |
| LGALS3BP | 2.04 | 4.60 |  |
| DACT1 | 2.07 | 2.00 |  |
| CALD1 | 2.11 | 2.30 |  |
| KCNMB1 | 2.12 |  | 6.71 |
| SEL1L3 (KIAA0746) | 2.16 | 2.30 |  |
| LIF | 2.18 | 4.90 |  |
| KIAA1671 | 2.25 | 6.30 |  |
| KRT7 | 2.29 | 3.70 |  |
| BST1 | 2.45 | 4.50 |  |
| C1orf54 (FLJ23221) | 2.48 | 4.50 |  |
| CDH6 | 2.49 | 3.70 |  |
| COL4A1 | 2.51 | 3.60 | 7.83 |
| JAG1 | 2.51 | 10.60 | 25.22 |
| SOBP (FLJ10159) | 2.65 | 4.00 |  |
| LPCAT2 (FLJ20481) | 2.67 | 2.00 |  |
| EGFLAM (FLJ39155) | 2.74 | 3.80 |  |
| INHBA | 2.75 |  | 9.93 |
| SDPR | 2.80 | 2.70 |  |
| CCDC81 (FLJ23514) | 2.81 | 4.10 |  |
| TPD52L1 | 2.85 | 3.20 |  |
| ACTA2 | 2.94 | 2.30 |  |
| RGS7 | 2.96 | 4.10 |  |
| RELN | 2.97 | 3.70 | 15.69 |
| F2R | 3.20 | 6.40 | 37.86 |
| LRRN3 | 3.21 | 2.40 |  |
| MRVI1 | 3.42 | 4.30 |  |
| OSBPL10 | 3.63 | 3.50 |  |
| LPPR4 | 4.16 |  | 17.95 |
| DMD | 4.19 | 5.10 | 13.13 |
| NTN4 | 4.87 | 15.50 |  |
| MEOX2 | 5.36 | 29.10 |  |
| EDIL3 | 5.45 | 4.00 |  |
| MEST | 5.80 | 14.40 | 19.34 |
| SRGN (PRG1) | 5.95 | 17.00 | 51.85 |
| F2RL2 | 6.43 | 4.80 |  |
| KRT18 | 6.68 | 12.80 | 17.74 |
| HHIP | 7.00 | 4.20 |  |

**B)** Genes with discordant (opposite) transcriptional patterns

| **Gene symbol** | **Fold change** | | |
| --- | --- | --- | --- |
|  | ***LMNA^K542/K542N^*** | ***LMNA^G608G/+^* [28]** | ***LMNA^G608G/+^* [29]** |
| TNFRSF11B | -2.26 | 3.00 |  |
| PROS1 | -1.90 | 2.20 |  |
| C11orf54 (PTD012) | -1.75 | 2.10 |  |
| CLDN11 | -1.61 | 4.20 |  |
| PLD1 | -1.53 | 4.70 |  |
| ASS1 (ASS) | 1.60 |  | -20.73 |
| KCTD12 | 1.89 |  | -10.90 |
| GALNT6 | 2.04 | -2.00 |  |
| CCDC85A (KIAA1912) | 2.25 | -3.50 |  |
| ENPP1 | 2.41 | -6.80 |  |
| VAT1L (KIAA1576) | 2.55 | -6.00 |  |
| MYBL1 | 2.82 | -2.20 |  |
| NRG1 | 2.85 | -3.80 |  |
| TFPI2 | 4.16 | -3.00 |  |
| MAB21L1 | 4.71 |  | -9.84 |

(A) genes with concordant (matching) transcriptional patterns, (B) genes with discordant (opposite) transcriptional patterns. Genes differentially expressed in *LMNA^K542/K542N^* fibroblasts are sorted by fold-change and followed by the G608G expression data published by Csoka et al. [28] and Wang et al. [29]. The former gene annotation used by Csoka et al. and/or Wang et al is indicated in brackets [28,29].
