# Supplementary material for: Discordant Gene Expression Signatures and Related Phenotypic Differences in Lamin A- and A/C-Related Hutchinson-Gilford Progeria Syndrome (HGPS)
Source: PLoS One. 2011 Jun 27;6(6):e21433. doi: 10.1371/journal.pone.0021433 (PMC3124505; doi:10.1371/journal.pone.0021433)
Supplement: Table S4 — Overlap between LMNA G608G transcription signatures. (DOC) [file pone.0021433.s006.doc]

**Table S4.** **Overlap between *LMNA* G608G transcription signatures.**

| **Gene symbol** | **Fold change** | | |
| --- | --- | --- | --- |
|  | ***LMNA^G608G/+^* [28]** | ***LMNA^G608G/+^* [29]** | ***LMNA^K542/K542N^*** |
| DPT | -5.00 | -31.06 | -3.36 |
| MXRA5 (DKFZp564I1922) | -3.40 | -24.84 | -2.76 |
| COL4A2 | 2.50 | 5.02 | 1.72 |
| COL4A1 | 3.60 | 7.83 | 2.51 |
| JAG1 | 10.60 | 25.22 | 2.51 |
| RELN | 3.70 | 15.69 | 2.97 |
| F2R | 6.40 | 37.86 | 3.2 |
| DMD | 5.10 | 13.13 | 4.19 |
| MEST | 14.40 | 19.34 | 5.8 |
| SRGN (PRG1) | 17.00 | 51.85 | 5.95 |
| KRT18 | 12.80 | 17.74 | 6.68 |
| GABBR2 (GPR51) | -8.20 | -10.37 |  |
| MMP3 | -6.70 | -67.36 |  |
| IL13RA2 | -5.40 | -32.16 |  |
| COL13A1 | -2.70 | -9.13 |  |
| PSG9 | -2.20 | -6.12 |  |
| GATA6 | 3.90 | 23.2 |  |
| ZNF423 (OAZ) | 5.20 | 6.61 |  |
| NID2 | 5.30 | 9.13 |  |
| PNMA2 | 8.60 | 13.32 |  |

For each gene the published expression fold-changes are given [28,29]. In addition, *LMNA* K542N expression data are presented for genes overlapping in all three studies. The former gene annotation used by Csoka et al. and/or Wang et al is indicated in brackets [28,29].
