# Supplementary material for: Discordant Gene Expression Signatures and Related Phenotypic Differences in Lamin A- and A/C-Related Hutchinson-Gilford Progeria Syndrome (HGPS)
Source: PLoS One. 2011 Jun 27;6(6):e21433. doi: 10.1371/journal.pone.0021433 (PMC3124505; doi:10.1371/journal.pone.0021433)
Supplement: Table S5 — Results of laboratory tests in the HGPS family carrying the LMNA K542N mutation. (A) Laboratory tests. (B) Oral glucose tolerance test (oGTT) (DOC) [file pone.0021433.s007.doc]

**Table S5.** **Results of laboratory tests in the HGPS family carrying the *LMNA* K542N mutation.**

**A) Laboratory tests**

|  | ***LMNA^K542/+^* carriers** | | | | | | ***LMNA^K542/K542N^* patients** | | |
| --- | --- | --- | --- | --- | --- | --- | --- | --- | --- |
| **Family members** | **Mother** | **Father** | | | **Sister** | | **A** | **E** | **R** |
| Age (years) | 41 | 46 | | 15 | | | 9 | 19 | 21 |
| Sex | F | M | | F | | | M | M | F |
| Fasting glucose (n.r.: 2.8 - 5.5 mmol/L) | 5.6 | 5.6 | | 4.9 | | | 4.9 | 4.4 | 4.7 |
| Fasting insulin (n.r.: 1.7 – 31.0 mU/L) | 4.6 | 7 | | 6.7 | | | 6.5 | 1.9 | 3 |
| Fasting C-peptide (n.r.: 0.26-0.63 nmol/L) | 0.35 | 0.49 | | 0.34 | | | 0.24 | 0.09 | 0.16 |
| Cholesterol (n.r.: < 5.0 mmol/L) | 3.6 | 4.8 | | 2.6 | | | 2.5 | 2.5 | 2.7 |
| Triglycerides (n.r.: <2.0 mmol/L) | 2.93 | 2.97 | | 1.16 | | | 1.52 | 1.02 | 1.18 |
| HDL cholesterol (n.r.: >1.0 mmol/L) | 0.73 | 0.91 | | 0.84 | | | 0.8 | 0.88 | 0.88 |
| Cholesterol/HDL (n.r.: < 5.0 mmol/mmol) | 4.9 | 5.3 | | 3.1 | | | 3.1 | 2.8 | 3.1 |
| LDL cholesterol (n.r.: <3.00 mmol/L) | 1.54 | 2.54 | | 1.23 | | | 1.01 | 1.16 | 1.28 |
| **Homeostatic model assessment** | | | | | | | | | |
| HOMA2-IR | 0.8 | | 1.1 | | | 0.7 | 0.5 | <0.4^†^ | <0.4^†^ |
| HOMA2-IS (%) | 161.7 | | 106.7 | | | 115 | 118.8 | 272.3 | 258.5 |
| HOMA2-B (%) | 63.8 | | 80.5 | | | 81.2 | 64.2 | <70.40^†^ | <61.7^†^ |

**B) Oral glucose tolerance test (oGTT)**

|  | ***LMNA^K542/+^* carriers** | | ***LMNA^K542/K542N^* patients** | |
| --- | --- | --- | --- | --- |
| **Family member** | **Mother** | **Father** | **A** | **E** |
| Age (years) | 42 | 47 | 10 | 20 |
| Sex | F | M | M | M |
| Body weight (kg) | 60 | 55 | 12 | 13 |
| Fasting insulin (n.r.: 2.6-24.9 mU/L) | 7.2 | 2.8 | 3.2 | 1.2 |
| 120 min insulin (n.r.: 16-166 mU/L) | 16.2 | 32.5 | 3.1 | 4.3 |
| Fasting glucose (n.r.: 3.9-6.1 mmol/L) | 6.4 | 6.0 | 4.3 | 4.2 |
| 120 min glucose (n.r.: 4.4-7.8 mmol/L) | 10.3 | 11.2 | 4.5 | 3.9 |
| Fasting C-peptide (n.r.: 0.26-0.63 nmol/L) | 0.42 | 0.44 | 0.22 | 0.08 |
| Osteocalcin (ug/L)* | 16 | 10.6 | 106 | 50.5 |
| **Homeostatic model assessment** | | | | |
| HOMA2-IR | 1 | 1 | 0.5 | <0.4^†^ |
| HOMA2-IS (%) | 100.5 | 251.5 | 248 | >276.6^†^ |
| HOMA2-B (%) | 55.4 | 65.5 | 77.5 | <78.6^†^ |
| **Insulin sensitivity indices derived from oGTT measurements** | | | | |
| Cederholm index | 5.99 | 4.71 | 28.39 | 35.16 |
| Matsuda index | 6.30 | 7.10 | 39.94 | 60.33 |

By means of the HOMA2 calculator, insulin resistance (HOMA2-IR) and beta cell function (HOMA2-B) were derived from fasting C-peptide concentrations, insulin sensitivity (HOMA2-IS) from fasting insulin values. Insulin sensitivity derived from oGTT was calculated as previously described [42,43]. ^†^ - since the HOMA2 model does not allow working with values outside the steady-state, the minimally required insulin and C-peptide values (2.9 and 0.22, respectively) were used. * - osteocalcin normal range for males: 14.0-42.0 ug/L, menopausal females: 15.0-46.0 ug/L, children: 24.0-70.0ug/L. “n.r.” – normal range.
